# Supplementary material for: Controlling enzyme hydrolysis of branched polymers synthesised using transfer-dominated branching radical telomerisation via telogen and taxogen selection
Source: Commun Chem. 2024 Sep 4;7:197. doi: 10.1038/s42004-024-01283-3 (PMC11372115; doi:10.1038/s42004-024-01283-3)
Supplement: Supplementary file 2 — Supplementary Information [file 42004_2024_1283_MOESM2_ESM.pdf]

Controlling enzyme hydrolysis of branched polymers synthesised using transfer-dominated branching radical telomerisation *via* telogen and taxogen selection.

Samuel Mckeating,[1] Oliver B. Penrhyn-Lowe,[1] Sean Flynn,[1] Savannah R. Cassin,[1] Sarah Lomas,[1] Christopher Fidge,[2] Paul Price,[2] Stephen Wright,[1] Pierre Chambon[1] and Steve P. Rannard[1,*]

[1]Department of Chemistry & Materials Innovation Factory, University of Liverpool, Crown Street, Liverpool L69 7ZD, UK. Correspondence and requests for materials should be addressed to S.P.R. (email: srannard@liverpool.ac.uk)

[2]Unilever R&D, Port Sunlight Laboratory, Quarry Road East, Bebington, Wirral, CH63 3JW, UK

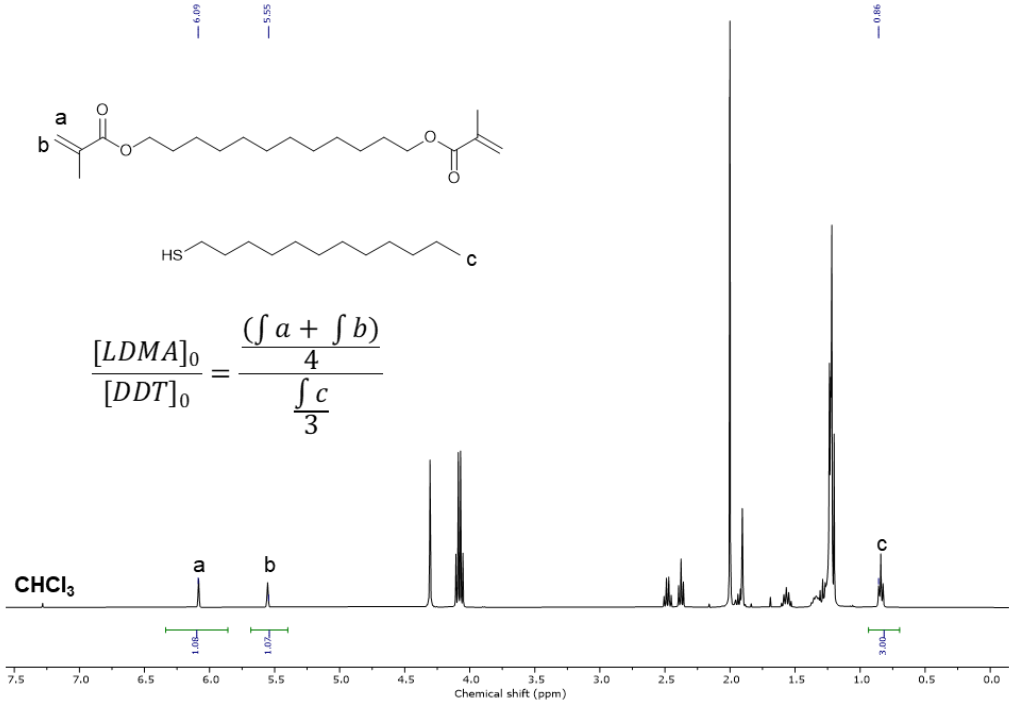

Figure S1 ^{1}H NMR (400 MHz, CDCl3) spectrum obtained from the homopolymerisation of LDMA with DDT prior to thermal initiation (t = 0). The equation provided was used to determine the initial ratio of LDMA to DDT ($[LDMA]_0/[DDT]_0$).

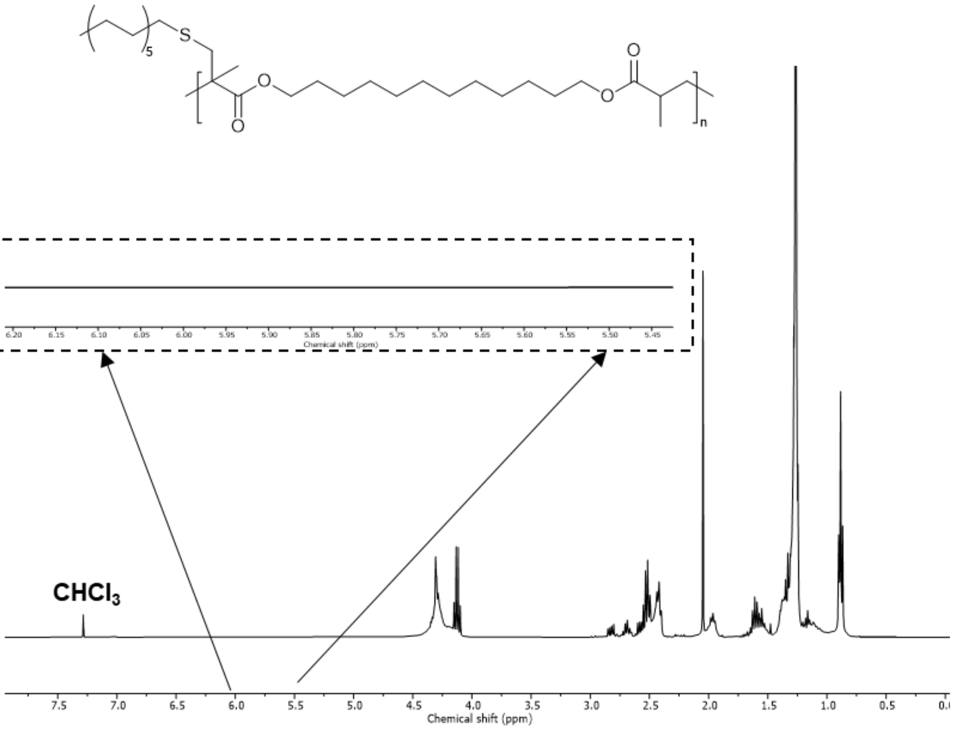

Figure S2 ^{1}H NMR (400 MHz, CDCl3) spectrum obtained from the homopolymerisation of LDMA with DDT ($[LDMA]_0/[DDT]_0 = 0.53$) at t = 24 hours. The total disappearance of vinyl signals at chemical shifts 5.50 ppm and 6.20 ppm indicate > 99 % vinyl conversion.

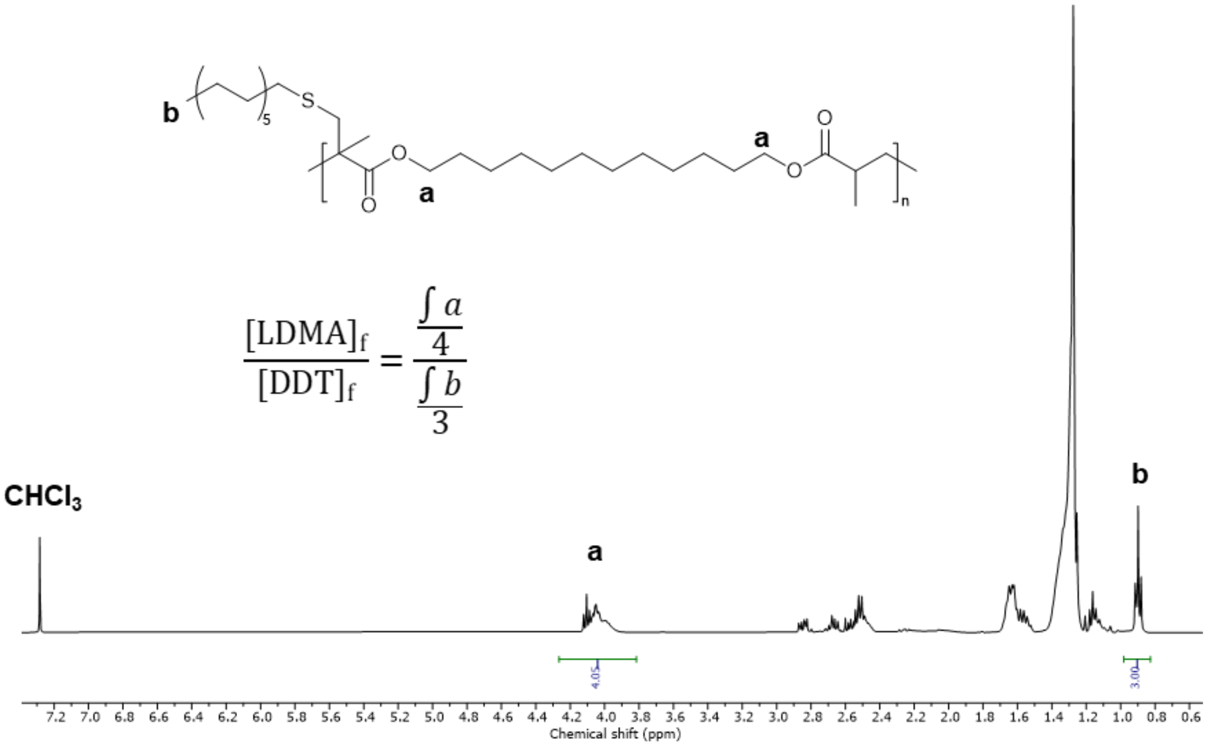

Figure S3 ^{1}H NMR (400 MHz, CDCl3) analysis of *p*(DDT-LDMA) demonstrating the calculation used to determine the final molar ratio of LDMA to DDT in the purified polymer ($[LDMA]_f/[DDT]_f$.

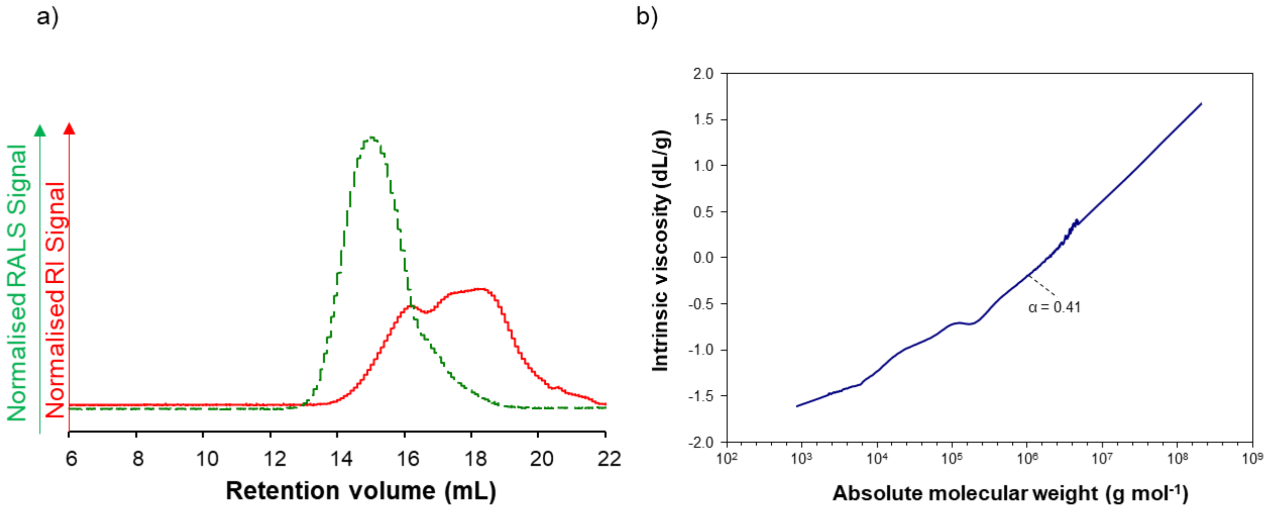

Figure S4 TD-SEC analysis of *p*(DDT-LDMA). a) Overlaid RI (red solid line) and RALS (green dashed line) chromatograms. B) b) evaluation of polymer topological structure *via* MHS analysis. $\alpha < 0.5$ indicates formation of a globular structure.

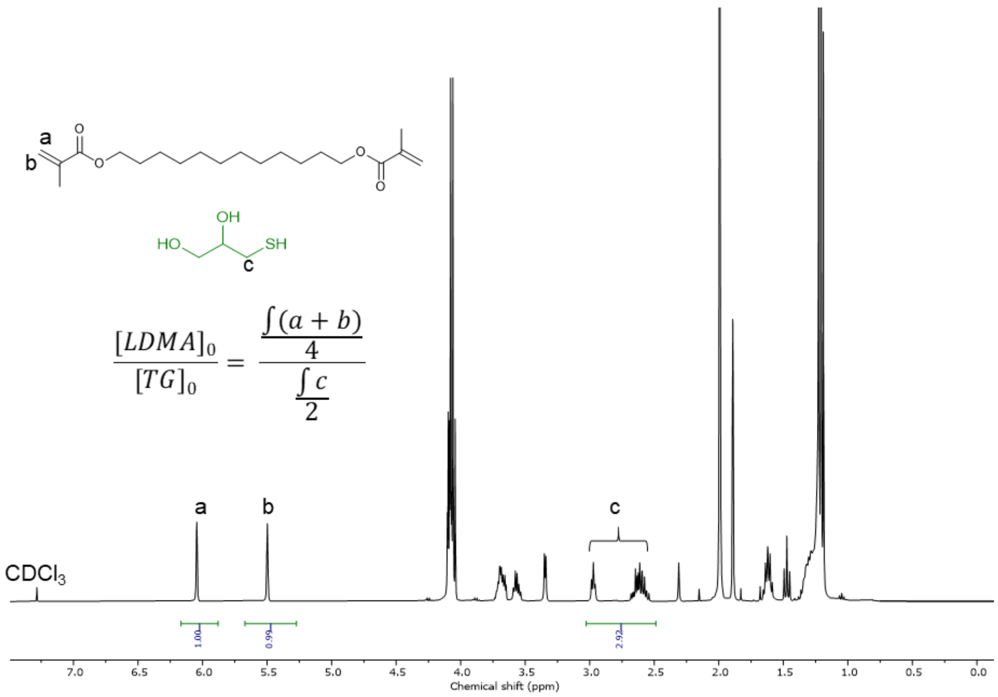

Figure S5 ^{1}H NMR (400 MHz, CDCl3) spectrum obtained from the homopolymerisation of LDMA with TG prior to thermal initiation (t = 0). The equation provided was used to determine the initial ratio of LDMA to TG ($[LDMA]_0/[TG]_0$).

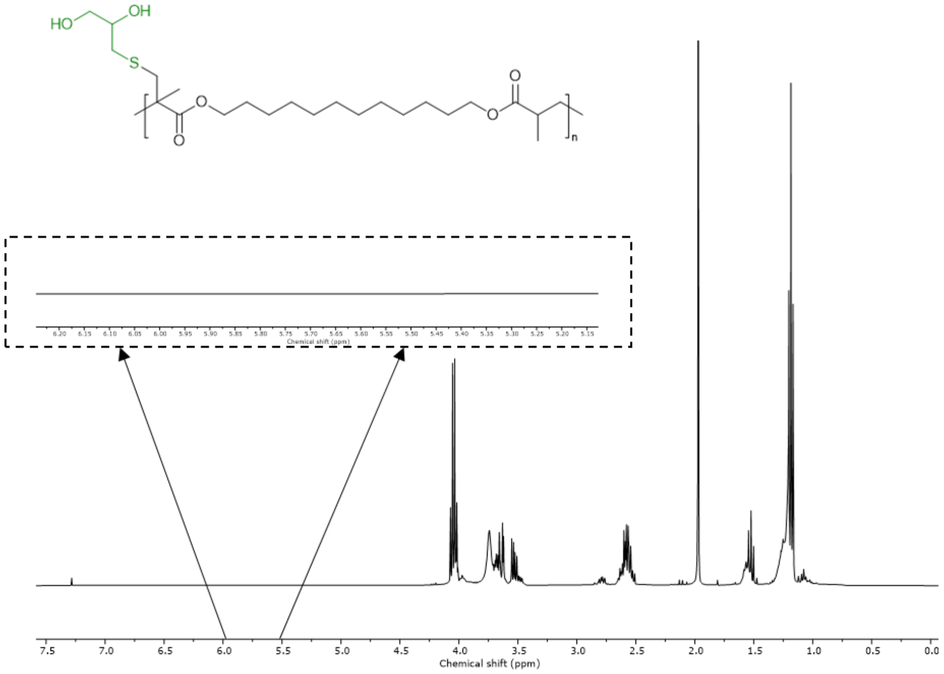

Figure S6 ^{1}H NMR (400 MHz, CDCl3) spectrum obtained from the homopolymerisation of LDMA with TG ($[LDMA]_0/[TG]_0 = 0.34$) at t = 24 hours. The total disappearance of vinyl signals at chemical shifts 5.50 ppm and 6.20 ppm indicate > 99 % vinyl conversion.

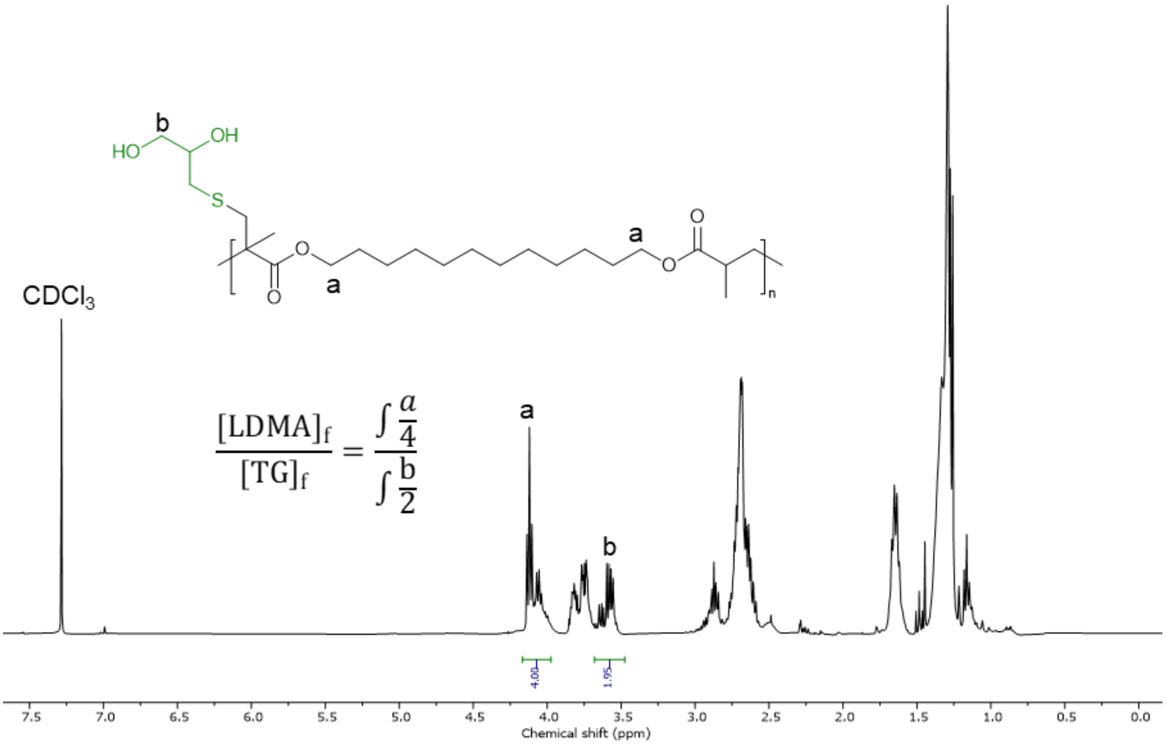

Figure S7 ^{1}H NMR (400 MHz, CDCl3) analysis of *p*(TG-LDMA) demonstrating the calculation used to determine the final molar ratio of LDMA to DDT in the purified polymer ($[LDMA]_f/[TG]_f$.

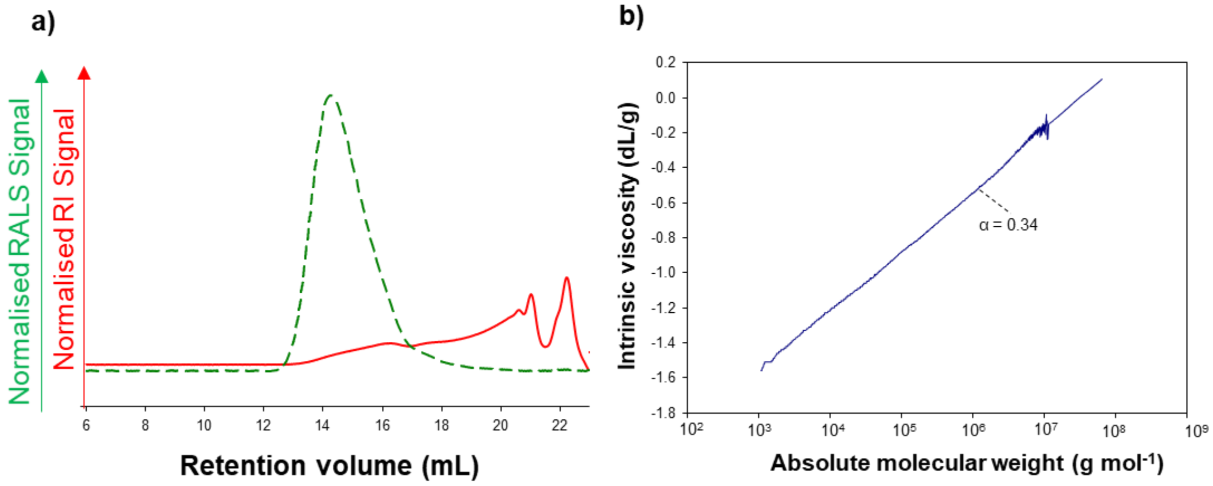

Figure S8 TD-SEC analysis of *p*(TG-LDMA). a) Overlaid RI (red solid line) and RALS (green dashed line) chromatograms. B) b) evaluation of polymer topological structure *via* MHS analysis. $\alpha < 0.5$ indicates formation of a globular structure.

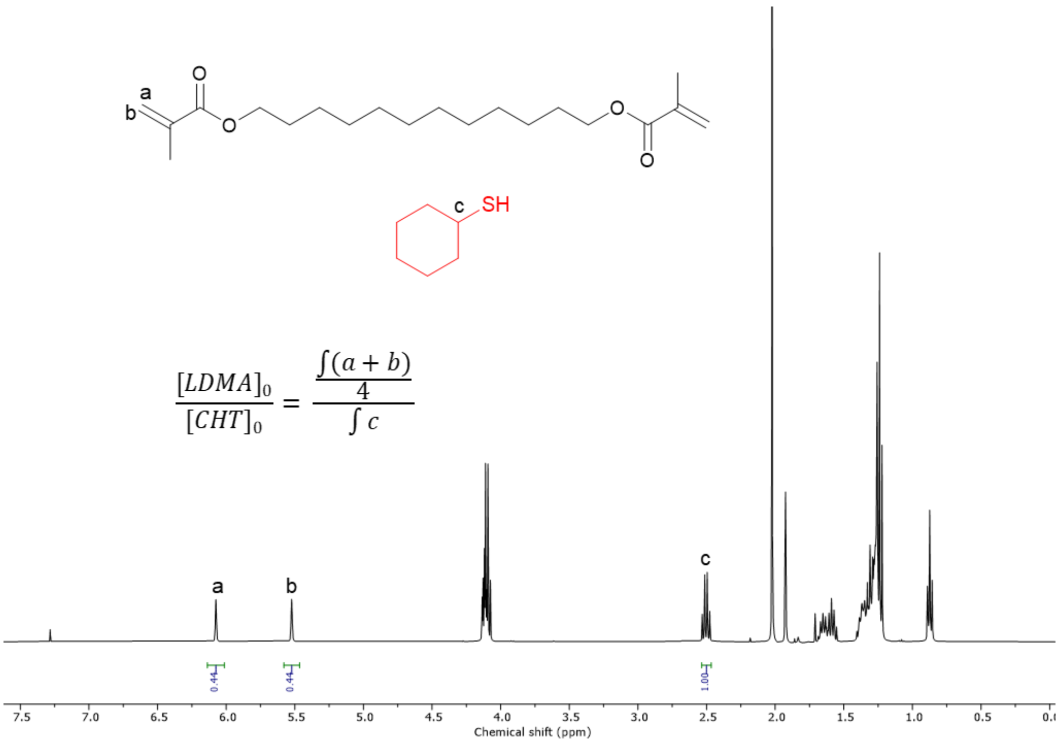

Figure S9 ^{1}H NMR (400 MHz, CDCl3) spectrum obtained from the homopolymerisation of LDMA with CHT prior to thermal initiation (t = 0). The equation provided was used to determine the initial ratio of LDMA to CHT ($[LDMA]_0/[CHT]_0$).

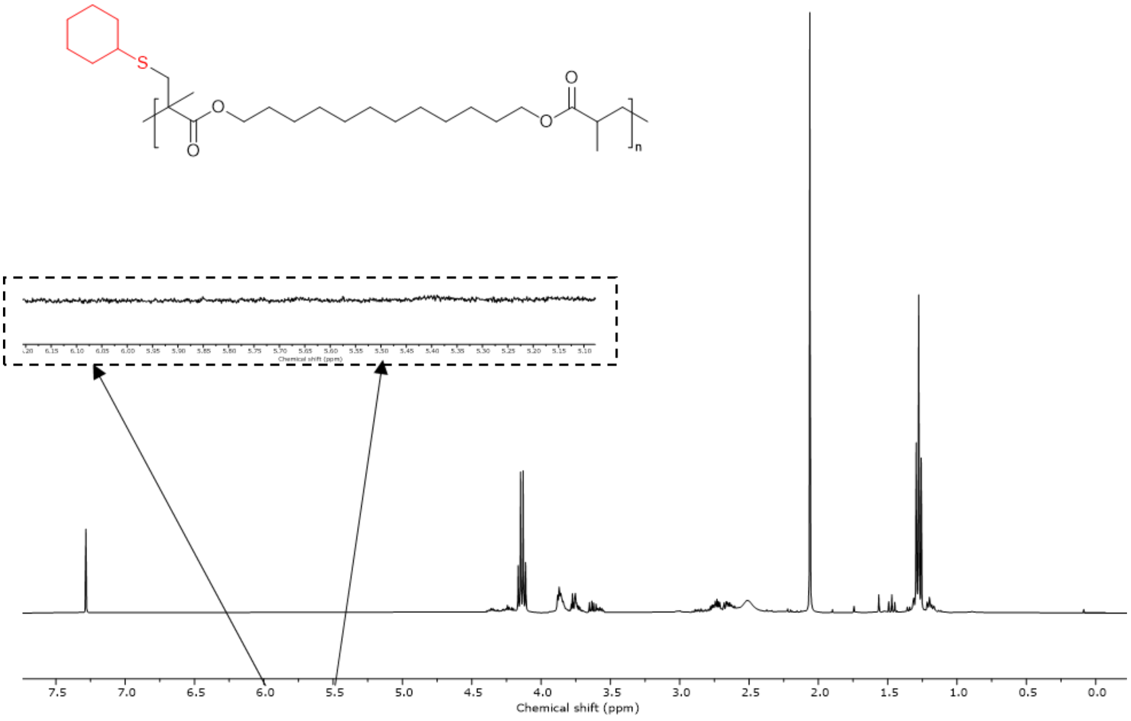

Figure S10 ^{1}H NMR (400 MHz, CDCl3) spectrum obtained from the homopolymerisation of LDMA with CHT ($[LDMA]_0/[CHT]_0$ = 0.22) at t = 24 hours. The total disappearance of vinyl signals at chemical shifts 5.50 ppm and 6.20 ppm indicate > 99 % vinyl conversion.

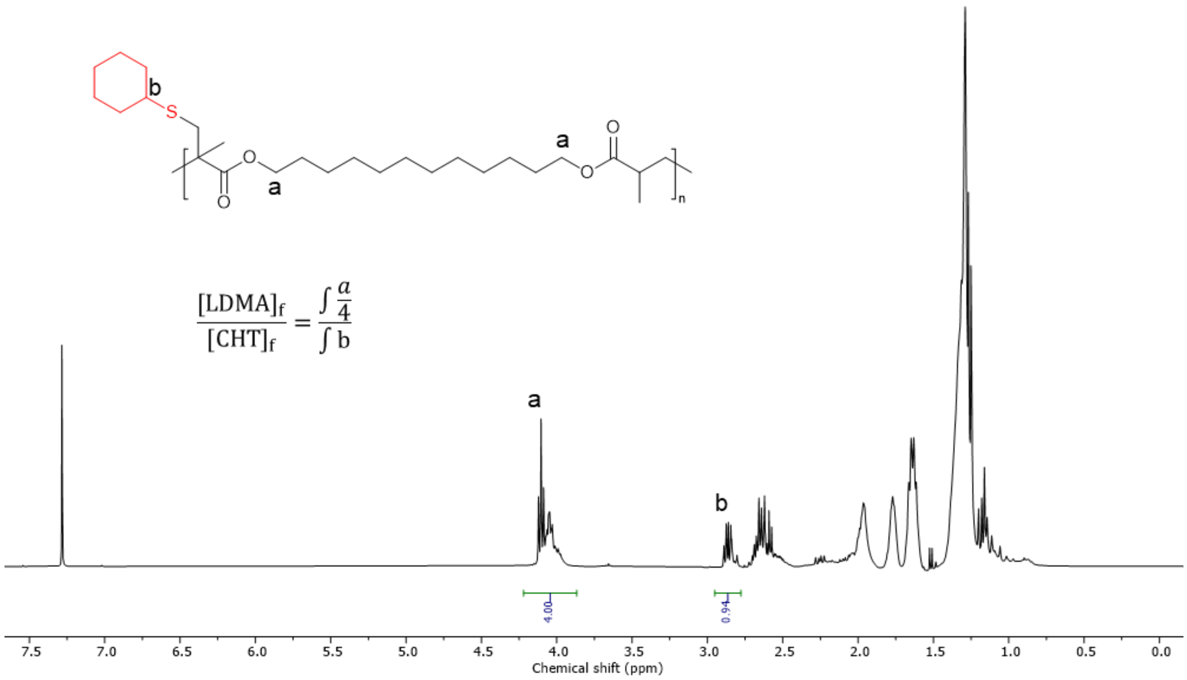

Figure S11 ^{1}H NMR (400 MHz, CDCl3) analysis of *p*(CHT-LDMA) demonstrating the calculation used to determine the final molar ratio of LDMA to CHT in the purified polymer ($[LDMA]_f/[CHT]_f$.

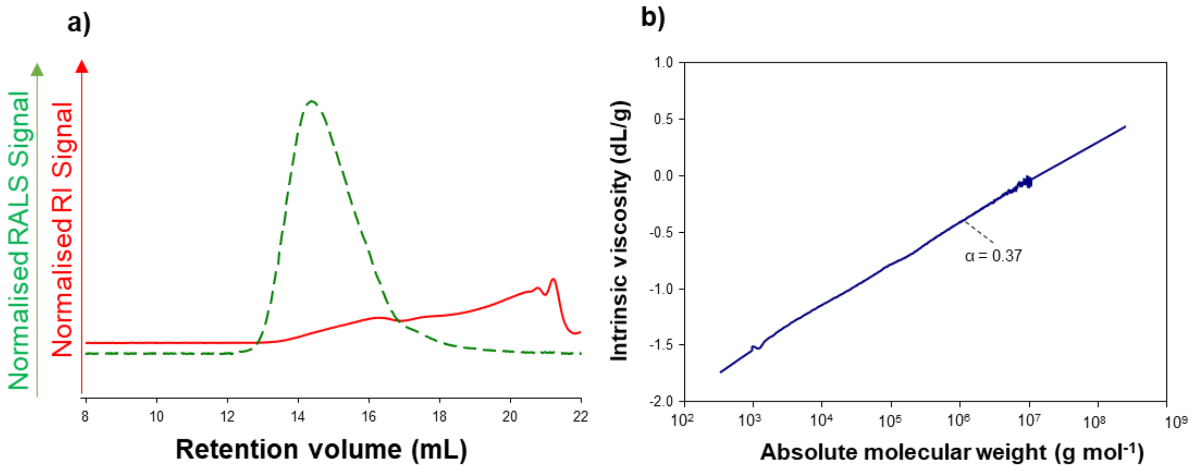

Figure S12 TD-SEC analysis of *p*(CHT-LDMA). a) Overlaid RI (red solid line) and RALS (green dashed line) chromatograms. B) b) evaluation of polymer topological structure *via* MHS analysis. $\alpha < 0.5$ indicates formation of a globular structure.

Table S1 Detailed characterisation by 1H NMR spectroscopy and TD-SEC of branched LDMA homopolymers synthesised with a range of telogens by TBRT

		^{1}H NMR				TD-SEC[d]			
**Taxogen**	**Telogen**	**$[MVT]_0/^a$ $[Tel]_0$**	**Conv.[b] (%)**	**$[EGDMA]_f$:[c] $[Tel]_f$**	**Gelation**	**M_w (g mol^{-1})**	**M_n (g mol^{-1})**	**Đ**	**α**
LDMA	DDT	0.4	> 99	0.87	N	11435	2320	4.9	0.354
LDMA	DDT	0.45	> 99	0.87	N	18912	3430	5.5	0.342
LDMA	DDT	0.49	> 99	0.85	N	36810	3210	11.4	0.361
LDMA	DDT	0.52	> 99	0.95	N	195526	14878	13.1	0.373
LDMA	DDT	0.54	> 99	1.01	N	249902	19483	12.8	0.354
LDMA	DDT	0.58	N/A	N/A	Y	N/A	N/A	N/A	N/A
LDMA	CHT	0.22	> 99	0.93	N	42206	3648	11.5	0.382
LDMA	CHT	0.25	> 99	0.94	N	55053	4450	12.4	0.336
LDMA	CHT	0.29	> 99	0.98	N	201205	6846	29.4	0.299
LDMA	CHT	0.31	> 99	0.95	N	310851	8006	38.8	0.33
LDMA	CHT	0.34	> 99	1.06	N	561087	10490	53.5	0.369
LDMA	CHT	0.37	N/A	N/A	Y	N/A	N/A	N/A	N/A
LDMA	TG	0.20	>99	1.11	N	15258	2046	7.5	0.347
LDMA	TG	0.25	>99	1.14	N	31387	4072	7.7	0.345
LDMA	TG	0.29	>99	0.97	N	59755	3925	15.2	0.345
LDMA	TG	0.32	>99	1.04	N	380492	13880	27.4	0.34
LDMA	TG	0.37	>99	1.04	N	893855	21224	42.1	0.311
LDMA	TG	0.39	N/A	N/A	Y	N/A	N/A	N/A	N/A

[a] Determined by ^{1}H NMR spectroscopy of the initial reaction mixture. [b] Determined by ^{1}H NMR spectroscopy of the crude reaction mixture taken at t = 24 hours. [c] Determined by ^{1}H NMR spectroscopy of the purified polymer. [d] Determined by TD-SEC of purified polymers using a THF/TEA mobile phase.

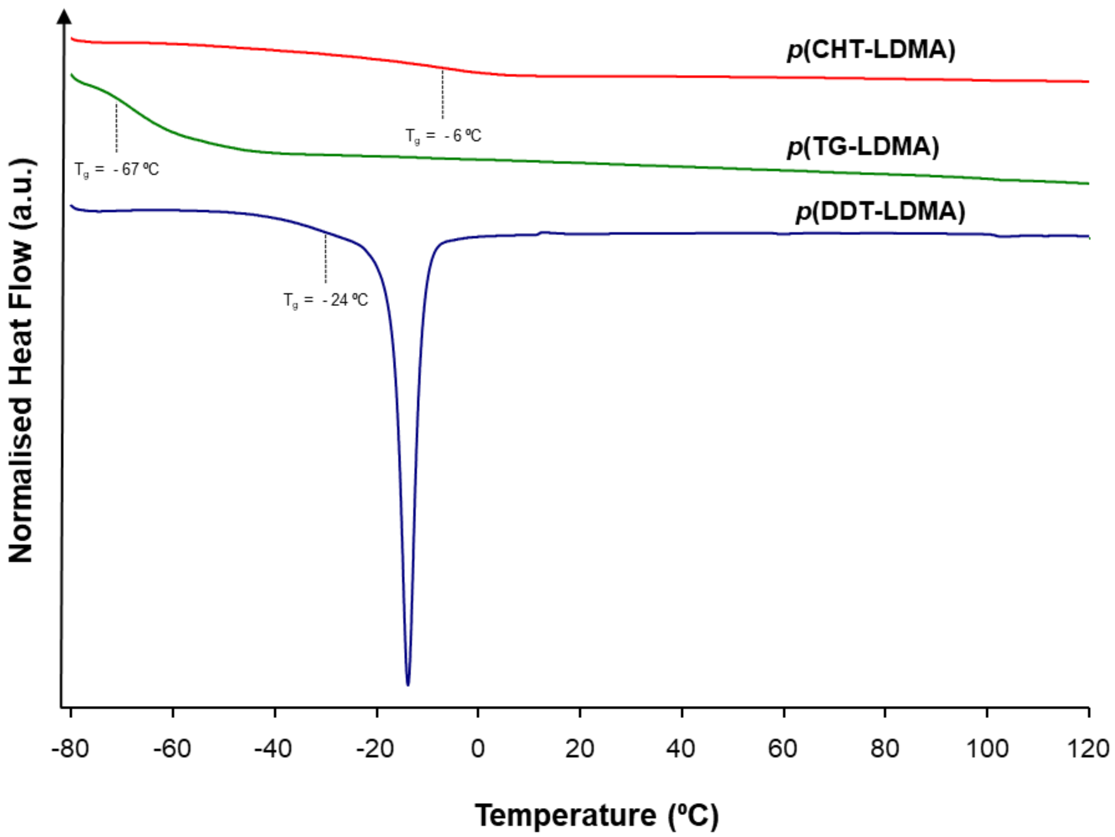

Figure S13 Overlaid thermograms obtained by DSC demonstrating the glass transition temperatures of three different telogen mediated LDMA TBRT polymers. Thermograms obtained for *p*(CHT-LDMA) (top, solid red line), *p*(TG-LDMA) (centre, solid green line) and *p*(DDT-LDMA) (bottom, solid blue line).

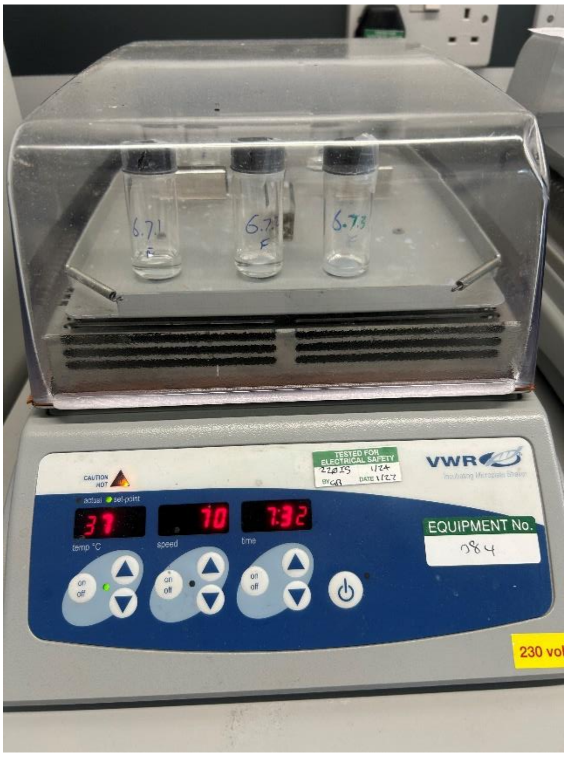

Figure S14 Photograph demonstrating the application of an incubating microplate shaker for use in hydrolytic degradation studies of polymer solutions at 37 ºC.

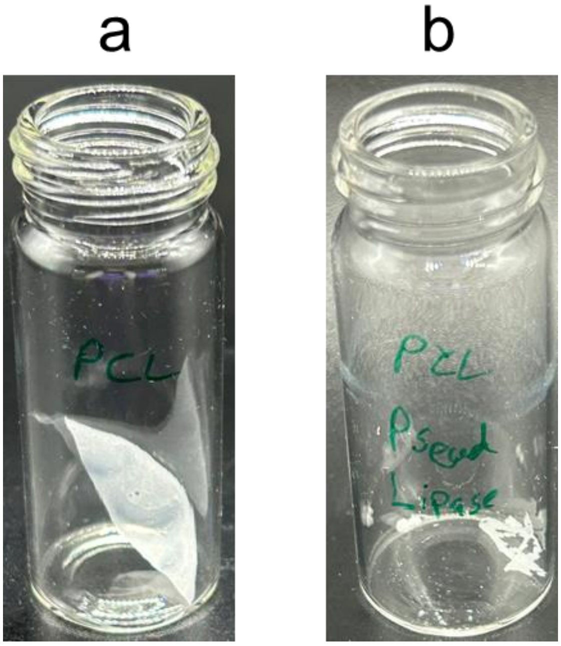

Figure S15 Photograph demonstrating the outcome of the hydrolytic degradation of a PCL film a) pre and b) post-exposure to the enzyme *Pseudomonas* lipase in Phosphate buffered saline at 37 ºC.

$$\text{Mass Remaining (\%)} = 100 - 100\left(\frac{\text{(Initial Polymer Mass} - \text{New Polymer Mass)}}{\text{Initial Polymer Mass}}\right) \qquad \textbf{Equation S1}$$

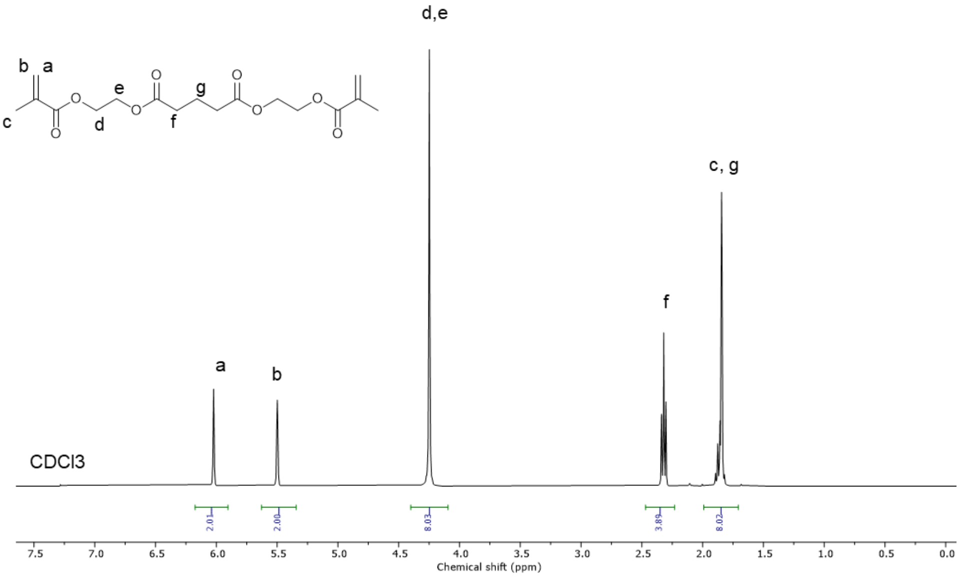

Figure S16 ¹H NMR (400 MHz, $CDCl_3$) spectrum obtained for purified bis-HEMA glutarate.

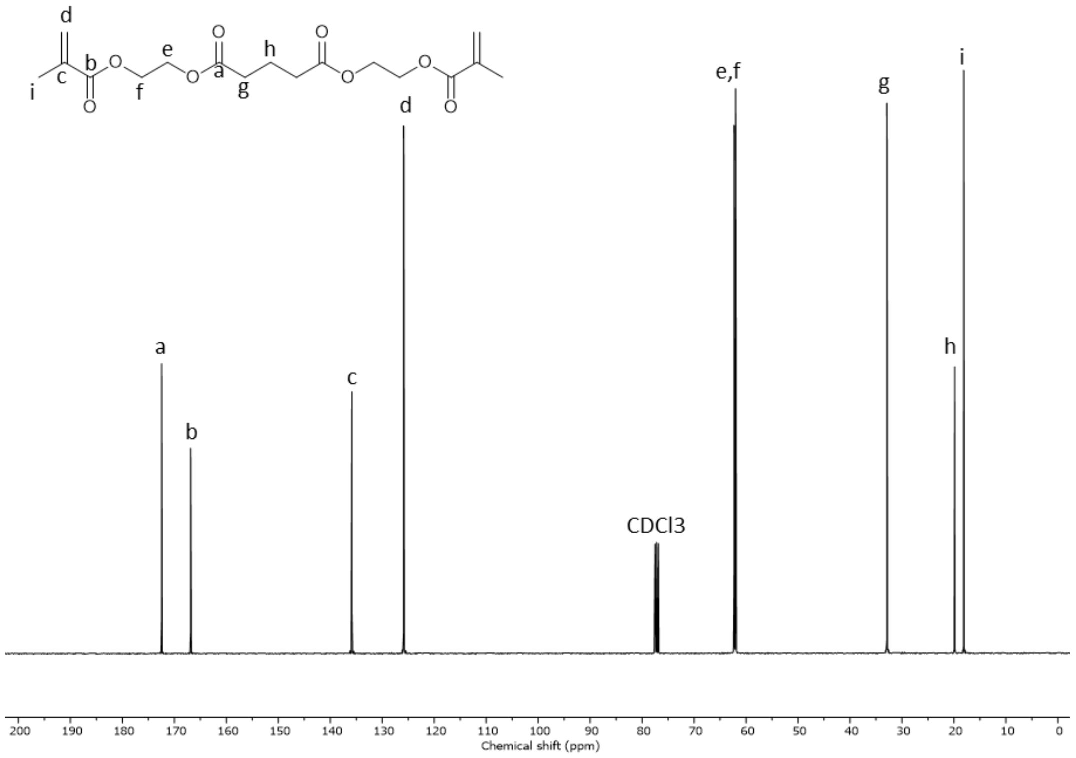

Figure S17 ¹³C NMR (400 MHz, $CDCl_3$) spectrum obtained for purified bis-HEMA glutarate.

Table S2 Elemental analysis of purified bis-HEMA glutarate

Sample Name	Empirical Formula	% Carbon		% Hydrogen		% Nitrogen	
		Calculated	Experimental	Calculated	Experimental	Calculated	Experimental
BHEMAG	$C_{17}O_8H_{24}$	57.3	57.4	6.8	6.8	0	0

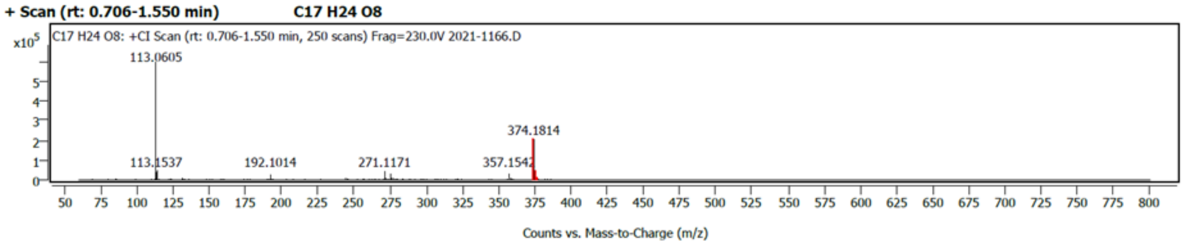

Spectrum Peaks

m/z	Z	Abund	Abund %	m/z (Calc)	Diff (ppm)	Ion Species	Formula	Ion Type
374.1814	1	202550	34.07	374.1809	1.12	(M+NH4)+	C17 H24 O8	
375.1842	1	35794	6.02	375.1842	-0.16	(M+NH4)+	C17 H24 O8	
376.1859	1	6443	1.08	376.1864	-1.44	(M+NH4)+	C17 H24 O8	

Figure S18 Chemical ionisation mass spectroscopy and tabulated mass list of purified bis-HEMA glutrate. Highest m/z demonstrated corresponds to bis-HEMA glutarate plus ammonia adduct (positive ion mode)

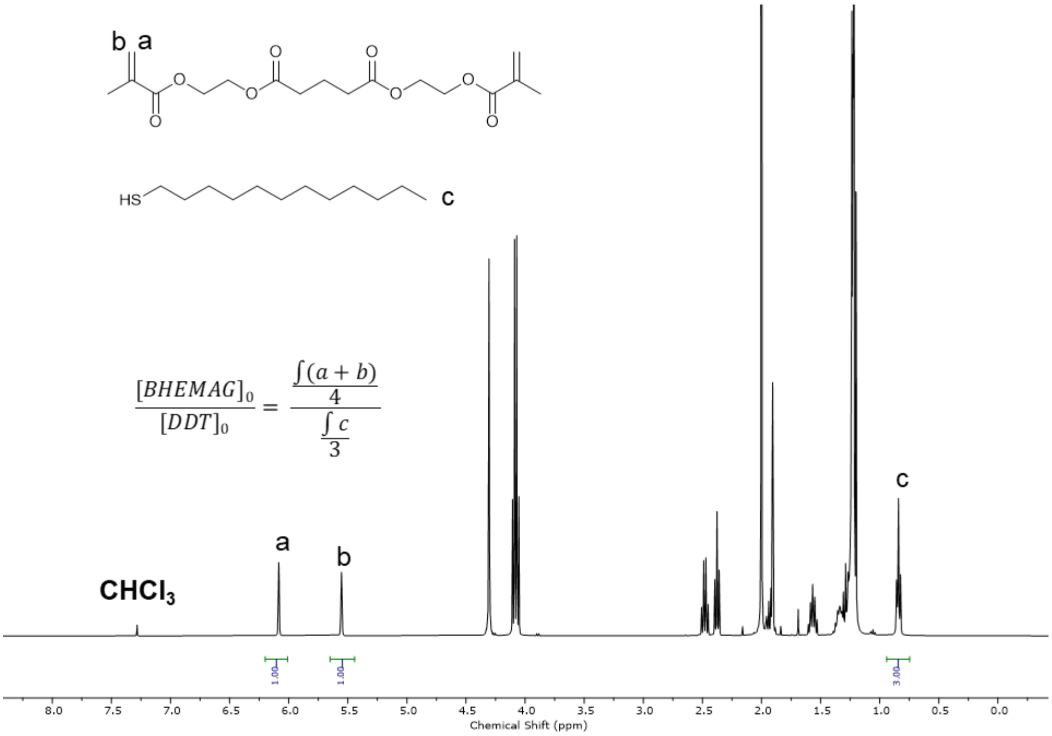

Figure S19 ^{1}H NMR (400 MHz, CDCl3) spectrum obtained from the homopolymerisation of BHEMAG with DDT prior to thermal initiation (t = 0). The equation provided was used to determine the initial ratio of BHEMAG to DDT ($[BHEMAG]_0/[DDT]_0$).

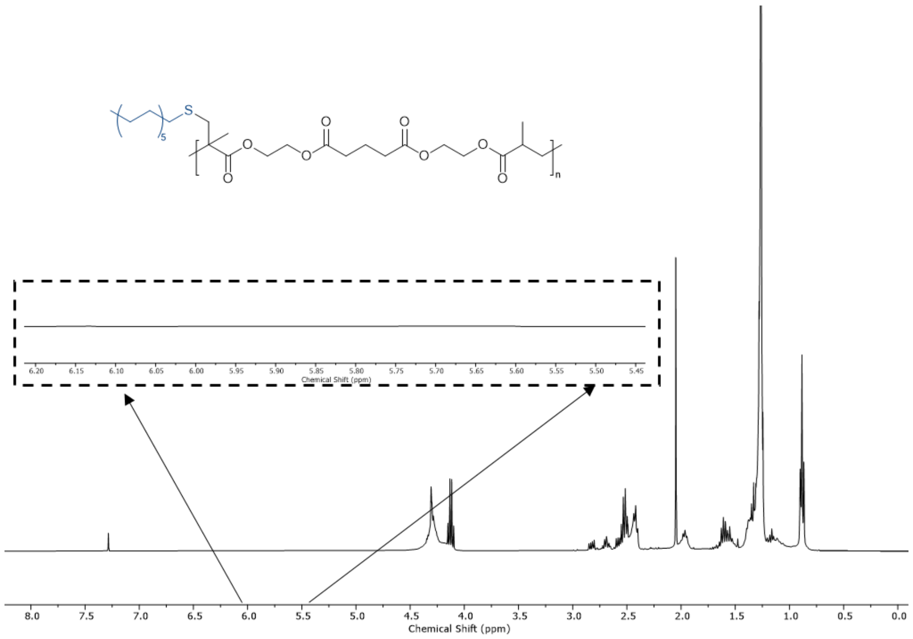

Figure S20 ^{1}H NMR (400 MHz, CDCl3) spectrum obtained from the homopolymerisation of BHEMAG with DDT ($[BHEMAG]_0/[DDT]_0$ = 0.50) at t = 24 hours. The total disappearance of vinyl signals at chemical shifts 5.50 ppm and 6.20 ppm indicate > 99 % vinyl conversion.

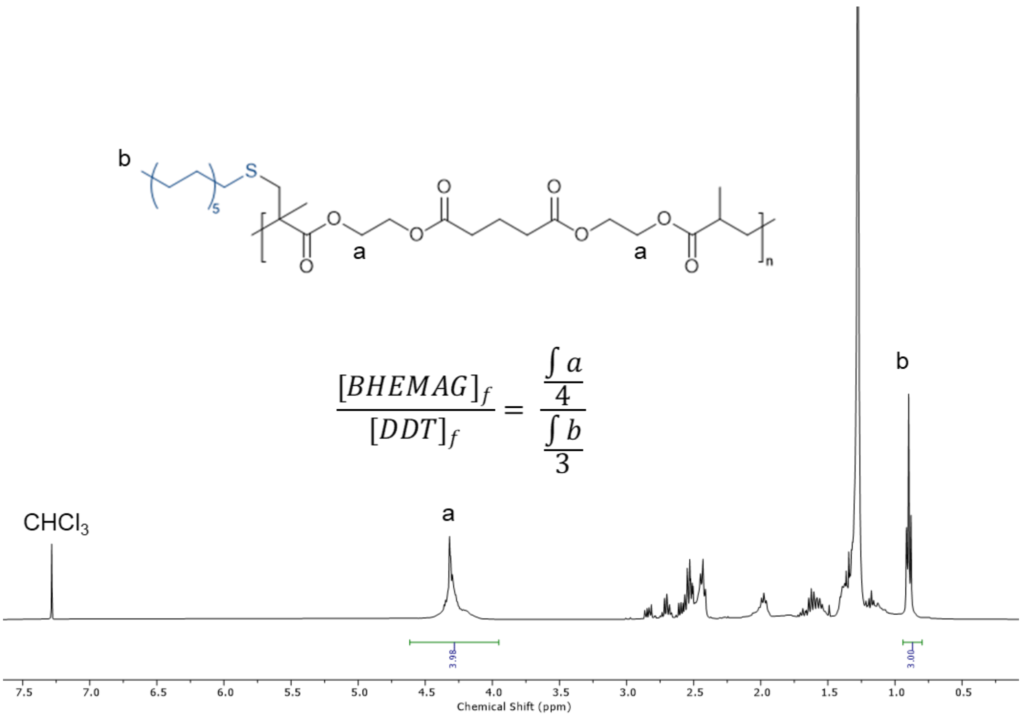

Figure S21 ^{1}H NMR (400 MHz, CDCl3) analysis of *p*(DDT-BHEMAG) demonstrating the calculation used to determine the final molar ratio of BHEMAG to DDT in the purified polymer ($[BHEMAG]_f/[DDT]_f$.

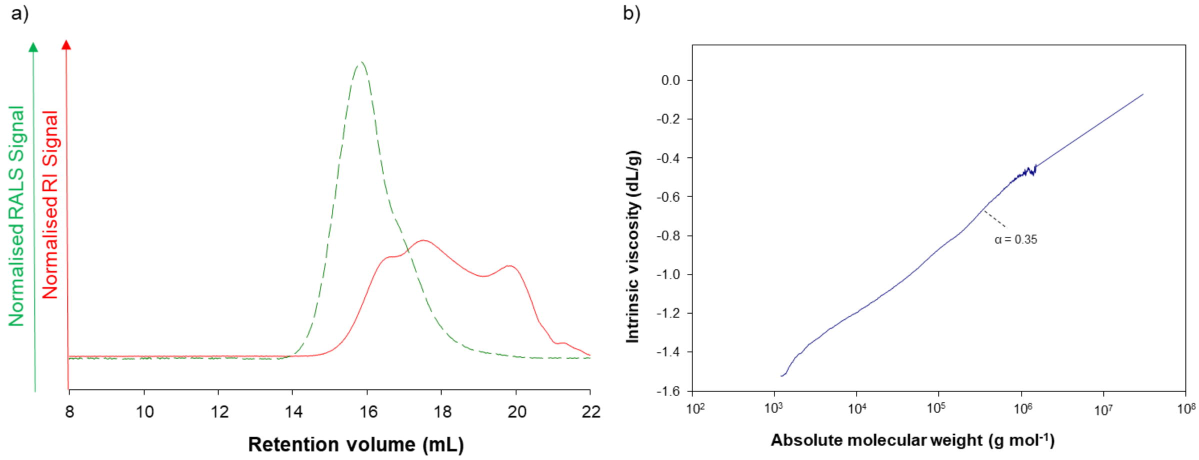

Figure S22 TD-SEC analysis of *p*(DDT-BHEMAG). a) Overlaid RI (red solid line) and RALS (green dashed line) chromatograms. B) b) evaluation of polymer topological structure *via* MHS analysis. $\alpha < 0.5$ indicates formation of a globular structure.

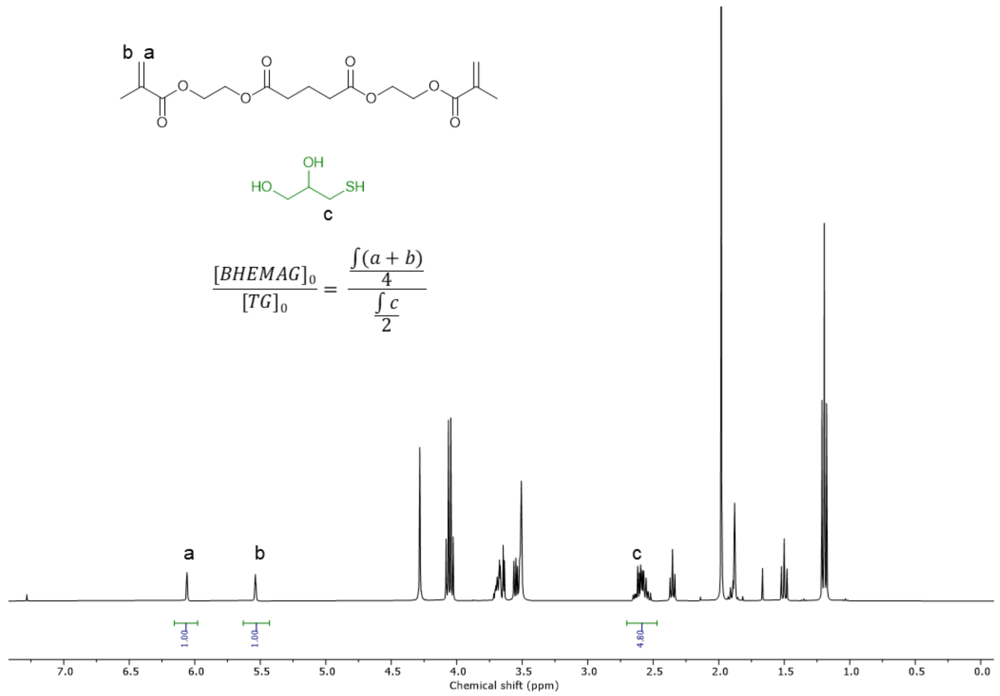

Figure S23 ^{1}H NMR (400 MHz, CDCl3) spectrum obtained from the homopolymerisation of BHEMAG with TG prior to thermal initiation (t = 0). The equation provided was used to determine the initial ratio of BHEMAG to TG ($[BHEMAG]_0/[TG]_0$).

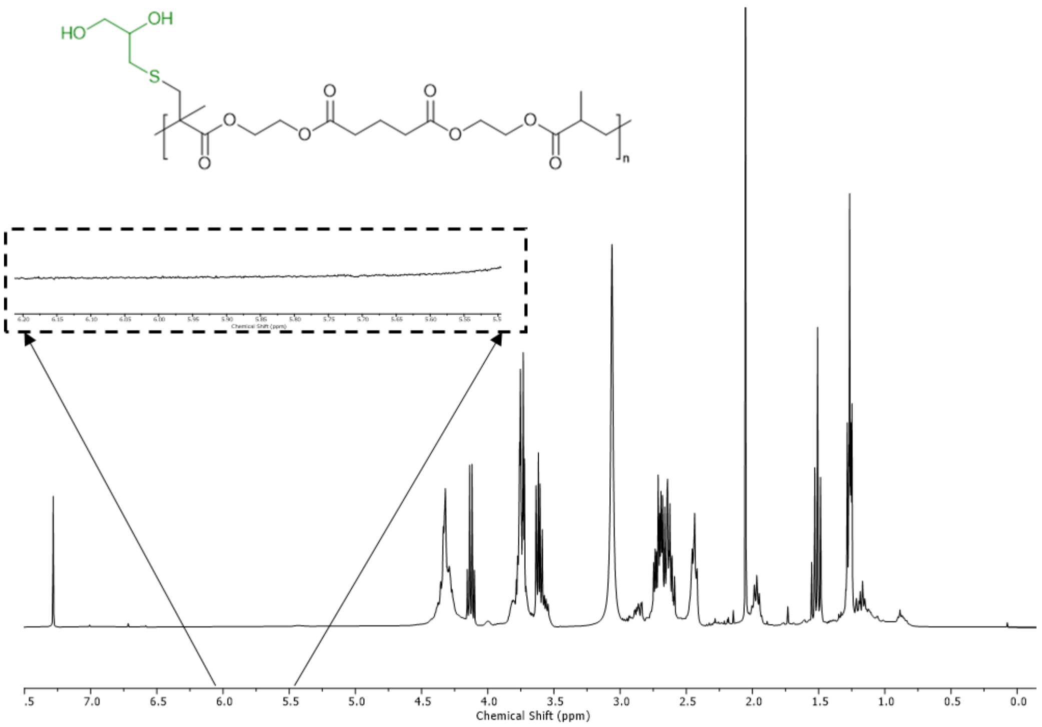

Figure S24 ^{1}H NMR (400 MHz, CDCl3) spectrum obtained from the homopolymerisation of BHEMAG with TG at t = 24 hours. The total disappearance of vinyl signals at chemical shifts 5.50 ppm and 6.20 ppm indicate > 99 % vinyl conversion.

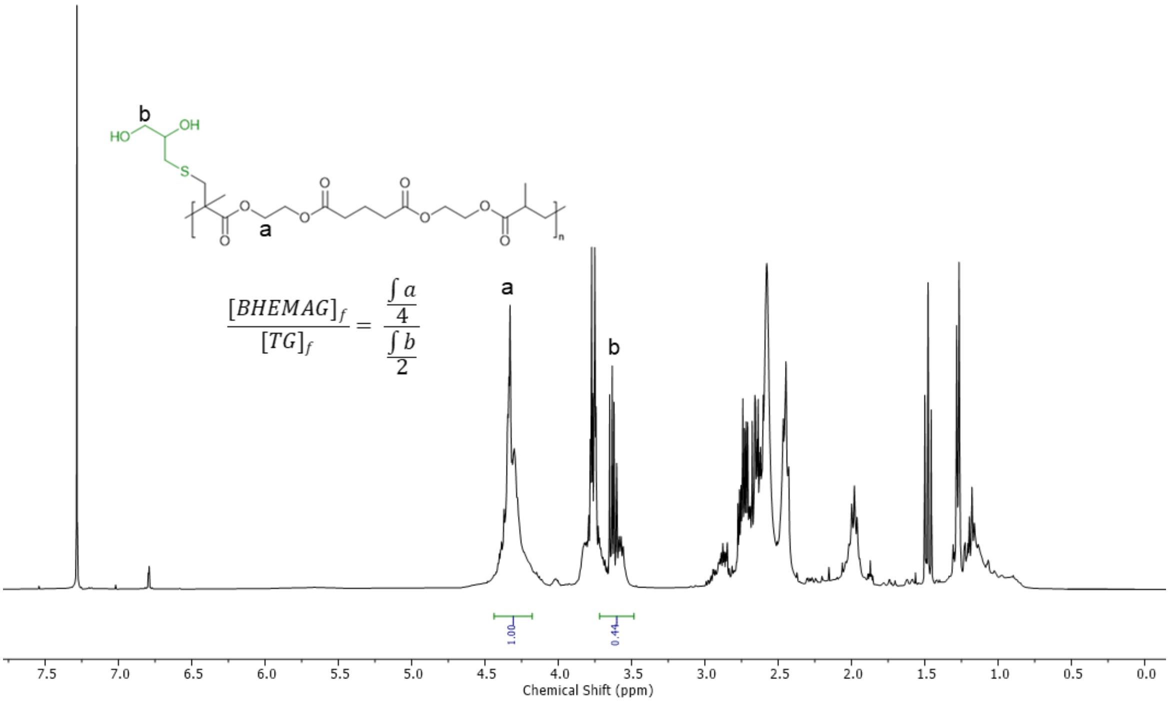

Figure S25 ^{1}H NMR (400 MHz, CDCl3) analysis of *p*(TG-BHEMAG) demonstrating the calculation used to determine the final molar ratio of BHEMAG to TG in the purified polymer ($[BHEMAG]_f/[TG]_f$.

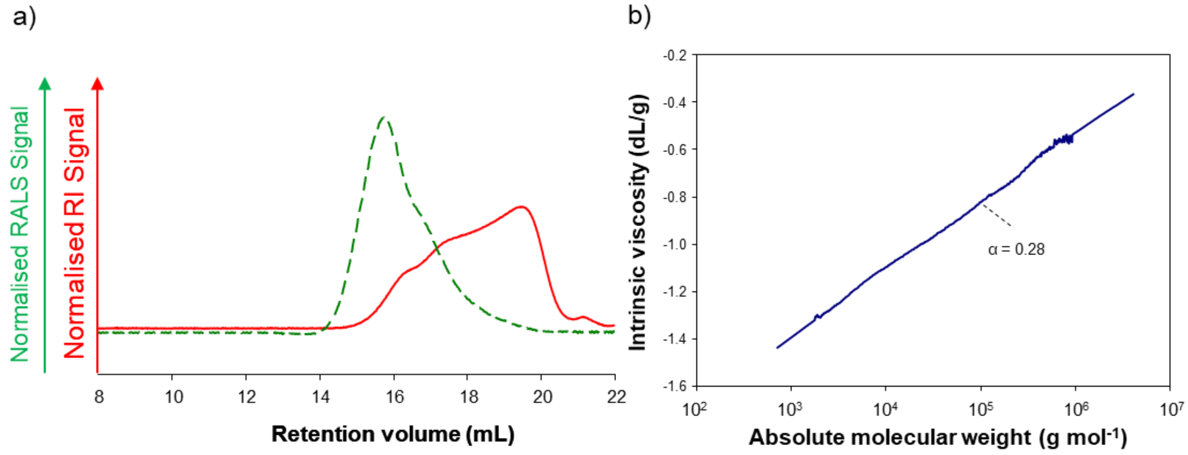

Figure S26 TD-SEC analysis of *p*(TG-BHEMAG). a) Overlaid RI (red solid line) and RALS (green dashed line) chromatograms. B) b) evaluation of polymer topological structure *via* MHS analysis. α < 0.5 indicates formation of a globular structure.

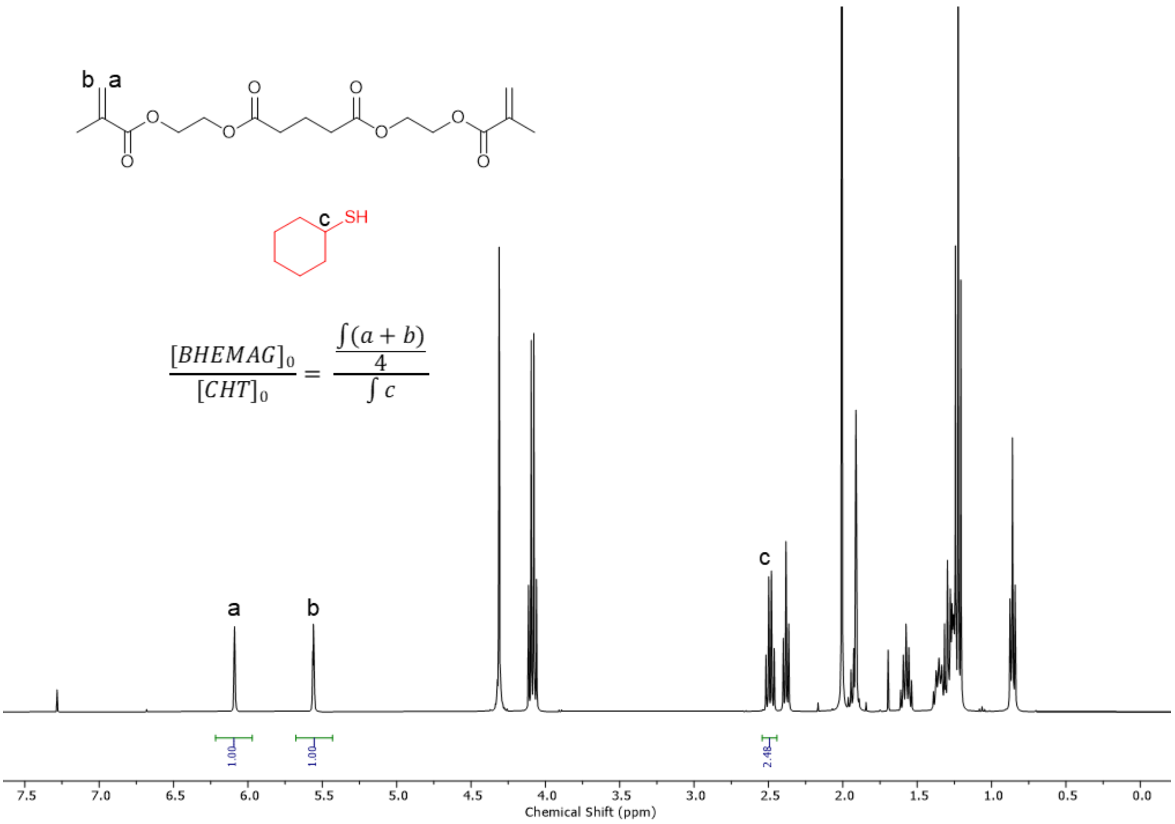

Figure S27 ^{1}H NMR (400 MHz, CDCl3) spectrum obtained from the homopolymerisation of BHEMAG with CHT prior to thermal initiation (t = 0). The equation provided was used to determine the initial ratio of BHEMAG to CHT ($[BHEMAG]_0/[CHT]_0$).

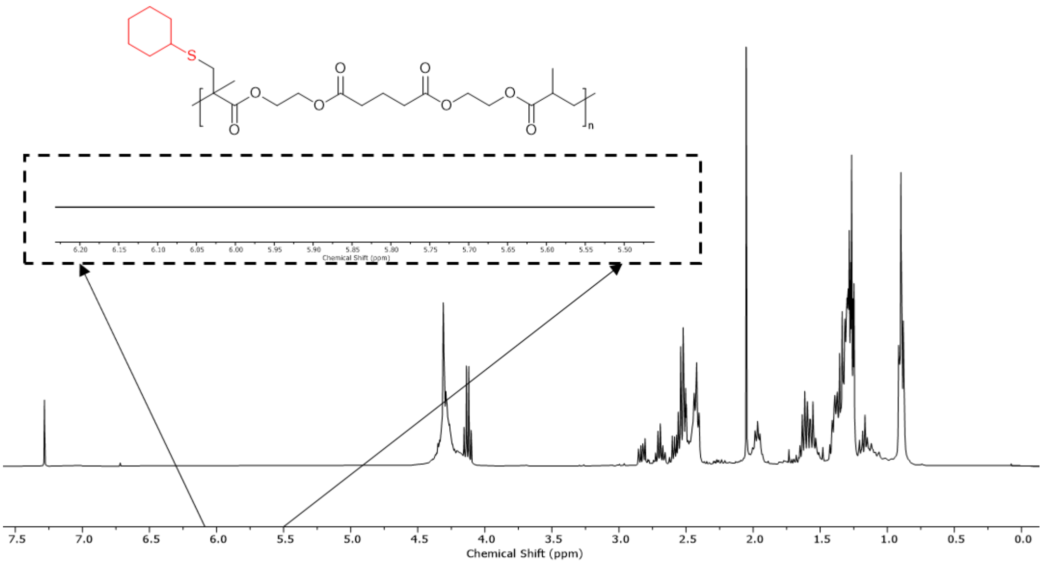

Figure S28 ^{1}H NMR (400 MHz, CDCl3) spectrum obtained from the homopolymerisation of BHEMAG with CHT at t = 24 hours. The total disappearance of vinyl signals at chemical shifts 5.50 ppm and 6.20 ppm indicate > 99 % vinyl conversion.

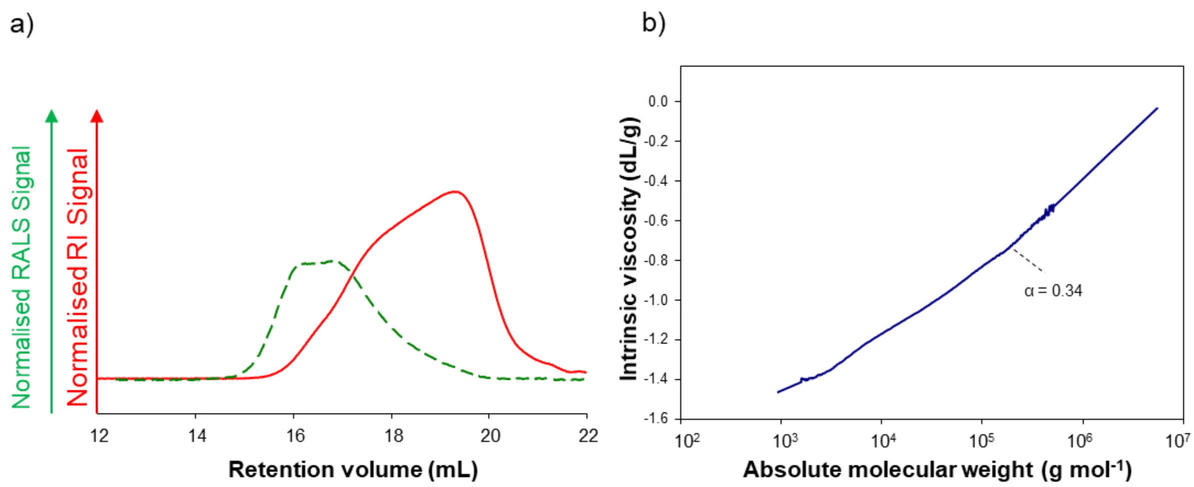

Figure S29 TD-SEC analysis of *p*(CHT-BHEMAG). a) Overlaid RI (red solid line) and RALS (green dashed line) chromatograms. B) b) evaluation of polymer topological structure *via* MHS analysis. α < 0.5 indicates formation of a globular structure.

Table S3 Detailed characterisation by 1H NMR spectroscopy and TD-SEC of branched BHEMAG homopolymers synthesised with a range of telogens by TBRT

		^{1}H NMR				TD-SEC[d]			
**Taxogen**	**Telogen**	**$[MVT]_0/[Tel]_0$[a]**	**Conv.[b] (%)**	**$[EGDMA]_f$:[Tel]$_f$[c]**	**Gelation**	**M_w (g mol^{-1})**	**M_n (g mol^{-1})**	**Đ**	**α**
BHEMAG	DDT	0.35	> 99	0.86	N	13629	829	16.5	0.279
		0.4	> 99	0.83	N	28743	1630	17.6	0.306
		0.46	> 99	0.89	N	39537	1155	34.2	0.328
		0.49	> 99	0.90	N	182119	6072	29.9	0.308
		0.55	> 99	1.03	N	234192	19629	11.9	0.35
		0.57	N/A	N/A	Y	N/A	N/A	N/A	N/A
BHEMAG	CHT	0.21	> 99	1.05	N	3718	1249	3.0	0.29
		0.25	> 99	0.95	N	8320	1705	4.9	0.428
		0.30	> 99	0.97	N	12323	3020	4.1	0.348
		0.32	> 99	1.00	N	57280	9668	5.9	0.331
		0.35	> 99	1.01	N	91778	12257	7.5	0.333
		0.38	N/A	N/A	Y	N/A	N/A	N/A	N/A
BHEMAG	TG	0.25	> 99	1.12	N	25938	3582	7.2	0.287
		0.3	> 99	1.00	N	37146	7697	4.8	0.387
		0.34	> 99	1.10	N	63186	8385	7.5	0.359
		0.36	> 99	1.06	N	271510	9436	28.8	0.303
		0.40	> 99	1.07	N	489489	14203	34.5	0.302
		0.42	N/A	N/A	Y	N/A	N/A	N/A	N/A

[a] Determined by ^{1}H NMR spectroscopy of the initial reaction mixture. [b] Determined by ^{1}H NMR spectroscopy of the crude reaction mixture taken at t = 24 hours. [c] Determined by ^{1}H NMR spectroscopy of the purified polymer. [d] Determined by TD-SEC of purified polymers using a THF/TEA mobile phase.

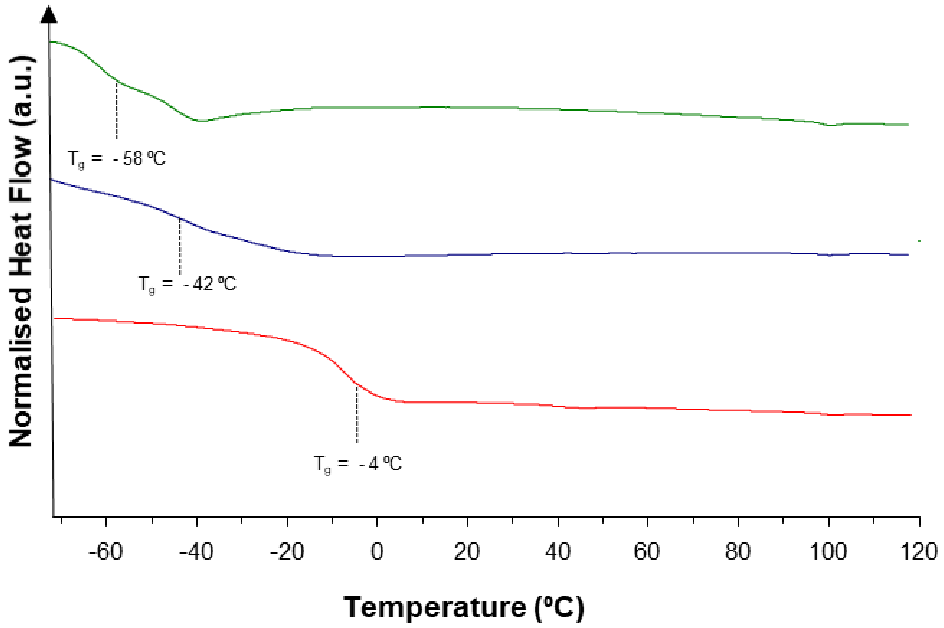

Figure S30 Overlaid thermograms obtained by DSC demonstrating the glass transition temperatures of three different telogen mediated BHEMAG TBRT polymers. Thermograms obtained for *p*(CHT-BHEMAG) (bottom, solid red line), *p*(TG-BHEMAG) (top, solid green line) and *p*(DDT-BHEMAG) (centre, solid blue line).

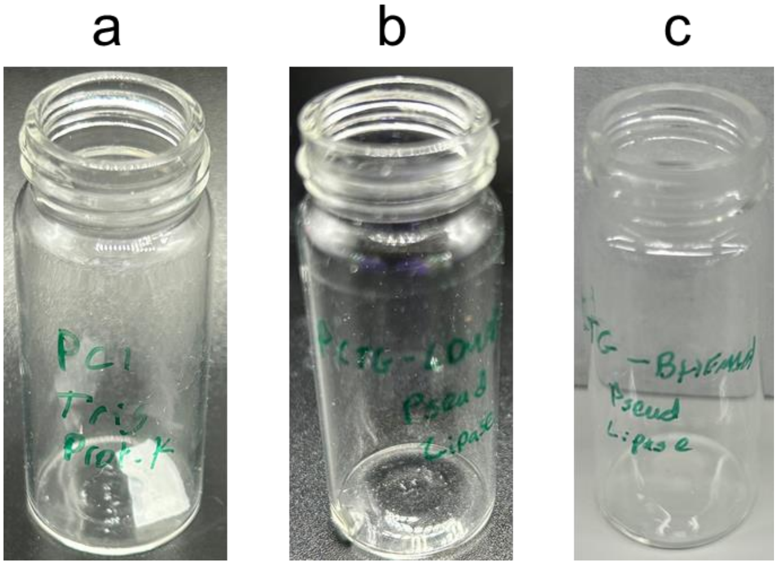

Figure S31 Photograph demonstrating the outcome of the hydrolytic degradation a) PCL film post exposure to Proteinase K, b) partially degraded p(TG-LDMA) film post exposure to *Pseudomonas* lipase and c) significantly degraded p(TG-BHEMAG) film post- exposure to the enzyme *Pseudomonas* lipase

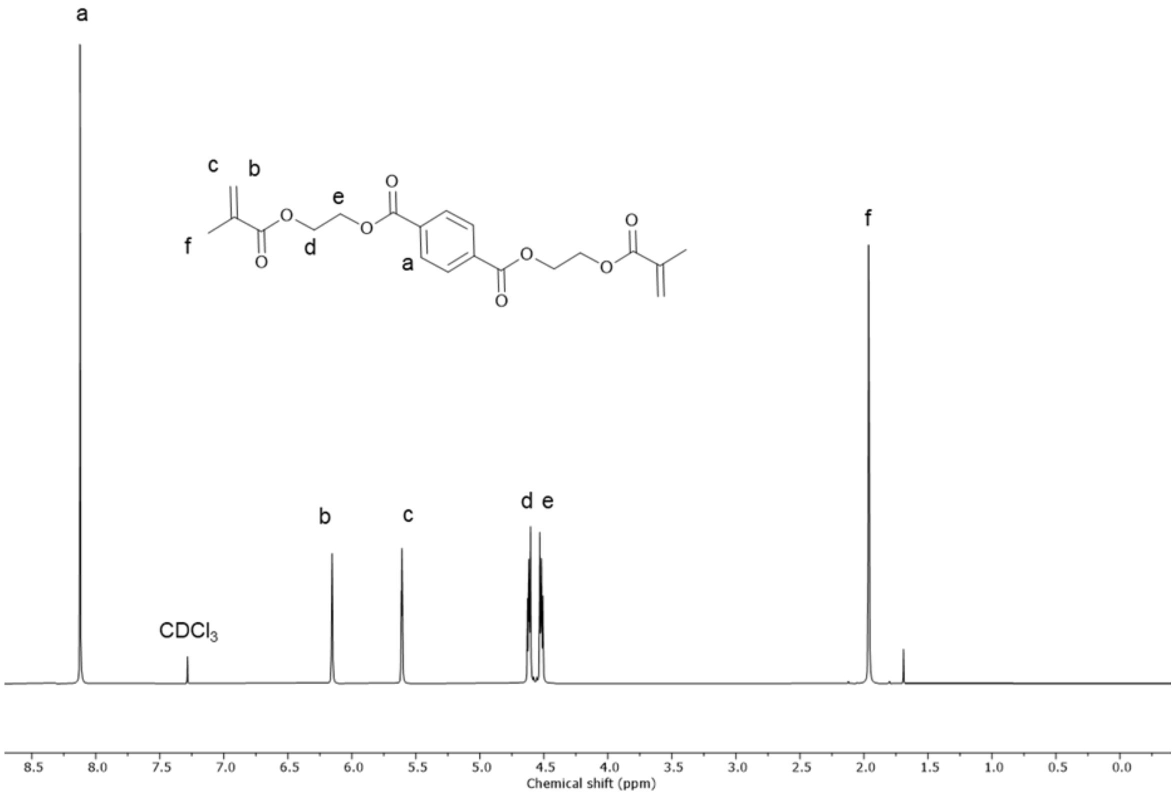

Figure S32 ^{1}H NMR (400 MHz, $CDCl_3$) spectrum obtained for purified bis-HEMA terephthalate

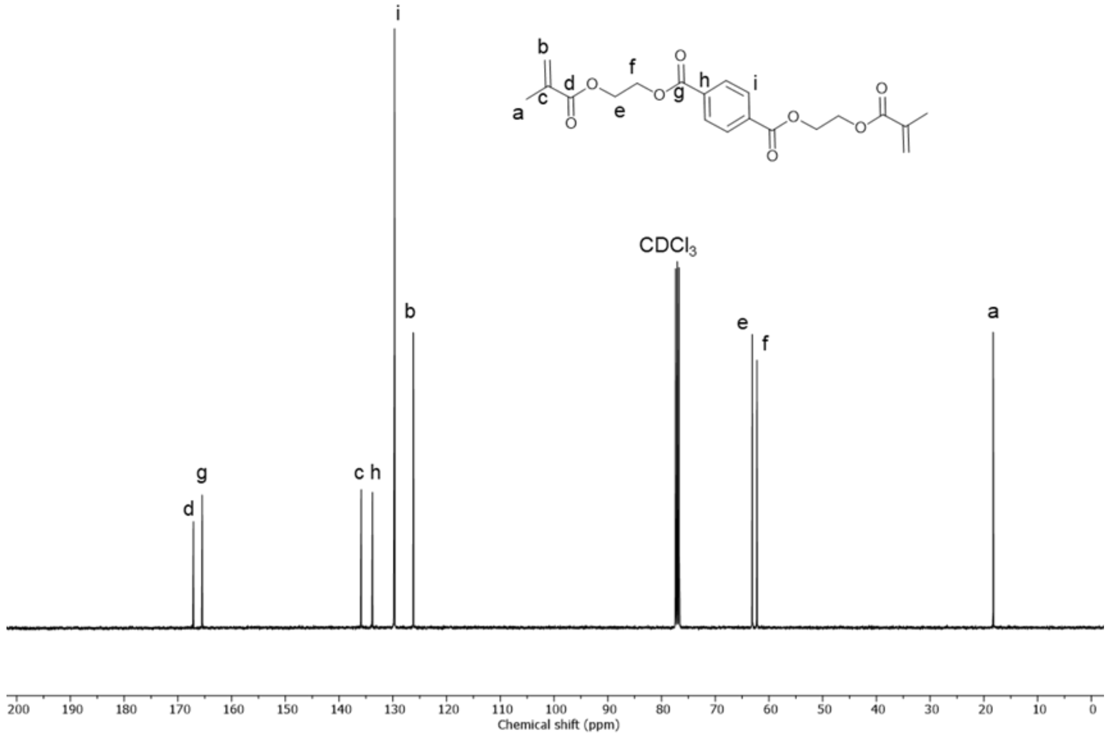

Figure S33 ^{13}C NMR (400 MHz, $CDCl_3$) spectrum obtained for purified bis-HEMA terephthalate

Table S4 Elemental analysis of purified bis-HEMA terephthalate

Sample Name	Empirical Formula	% Carbon		% Hydrogen		% Nitrogen	
		Calculated	Experimental	Calculated	Experimental	Calculated	Experimental
BHEMATP	$C_{20}O_8H_{22}$	61.5	61.3	5.7	6.0	0	0

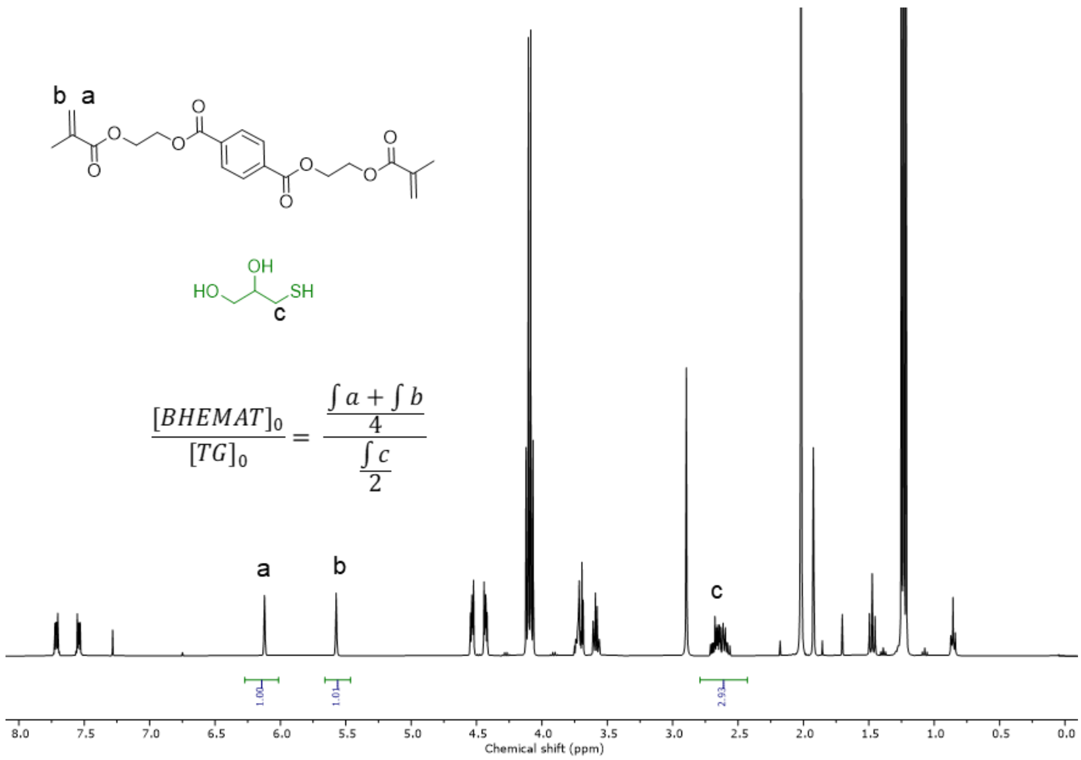

Figure S34 ^{1}H NMR (400 MHz, CDCl3) spectrum obtained from the homopolymerisation of BHEMAT with TG prior to thermal initiation (t = 0). The equation provided was used to determine the initial ratio of BHEMAT to TG ($[BHEMAT]_0/[TG]_0$).

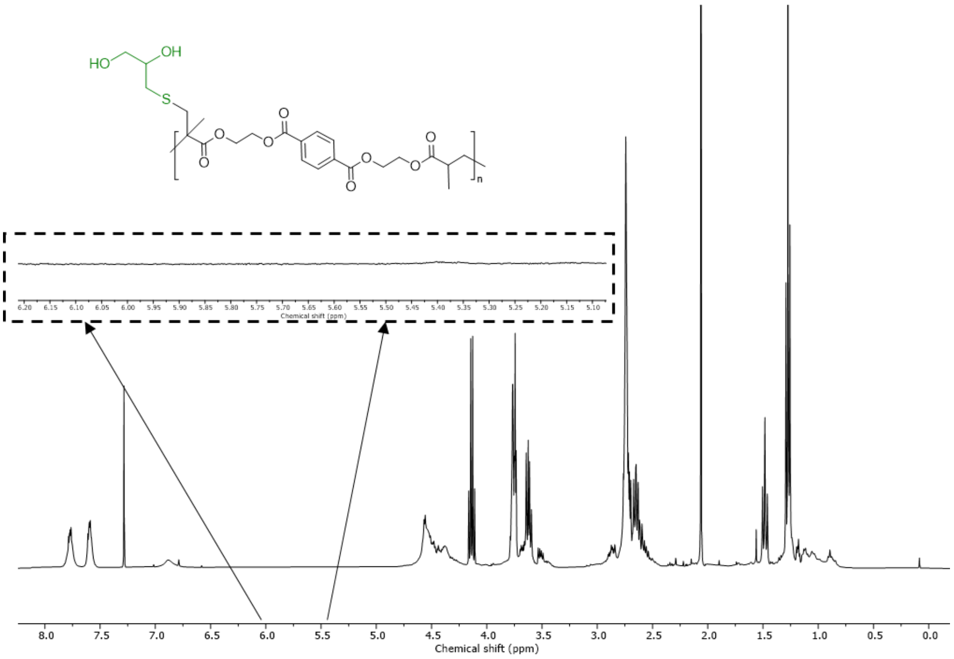

Figure S35 ^{1}H NMR (400 MHz, CDCl3) spectrum obtained from the homopolymerisation of BHEMAT with TG ($[BHEMAT]_0/[TG]_0 = 0.33$) at t = 24 hours. The total disappearance of vinyl signals at chemical shifts 5.50 ppm and 6.20 ppm indicate > 99 % vinyl conversion.

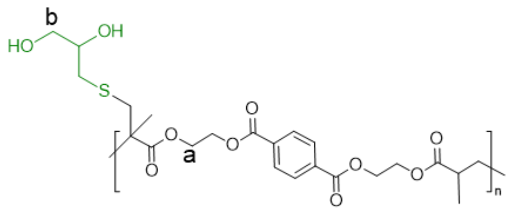

$$\frac{[BHEMAT]_f}{[TG]_f} = \frac{\frac{\int a}{4}}{\frac{\int b}{2}}$$

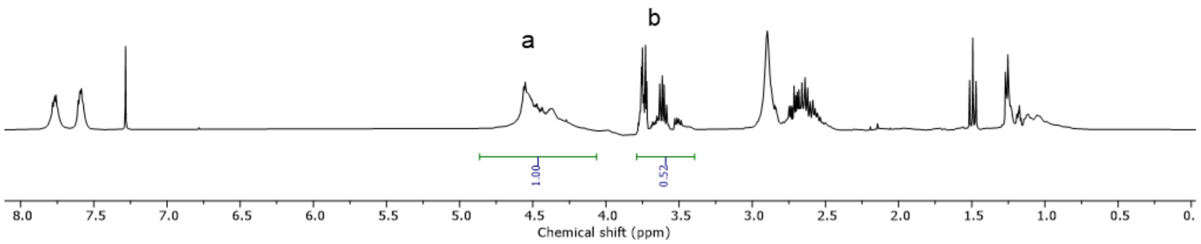

Figure S36 ^{1}H NMR (400 MHz, CDCl3) analysis of *p*(TG-BHEMAT) demonstrating the calculation used to determine the final molar ratio of BHEMAT to TG in the purified polymer ($[BHEMAT]_f/[TG]_f$.

Table S5 Detailed characterisation by 1H NMR spectroscopy and TD-SEC of branched BHEMAT homopolymers synthesised with 1-thioglycerol by TBRT

		^{1}H NMR				TD-SEC[d]			
Taxogen	Telogen	$[MVT]_0$/[a] $[Tel]_0$	Conv.[b] (%)	$[EGDMA]_f$:[c] $[Tel]_f$	Gelation	M_w (g mol^{-1})	M_n (g mol^{-1})	Đ	α
BHEMAT	TG	0.31	>99	0.96	N	14312	1466	9.8	0.403
		0.33	>99	0.96	N	17080	2805	6.1	0.386
		0.35	>99	1.02	N	23128	5174	4.5	0.326
		0.39	>99	0.94	N	62950	8073	7.8	0.401
		0.42	>99	1.00	N	236942	18625	12.7	0.301
		0.44	N/A	N/A	Y	N/A	N/A	N/A	N/A

[a] Determined by ^{1}H NMR spectroscopy of the initial reaction mixture. [b] Determined by ^{1}H NMR spectroscopy of the crude reaction mixture taken at t = 24 hours. [c] Determined by ^{1}H NMR spectroscopy of the purified polymer. [d] Determined by TD-SEC of purified polymers using a THF/TEA mobile phase.

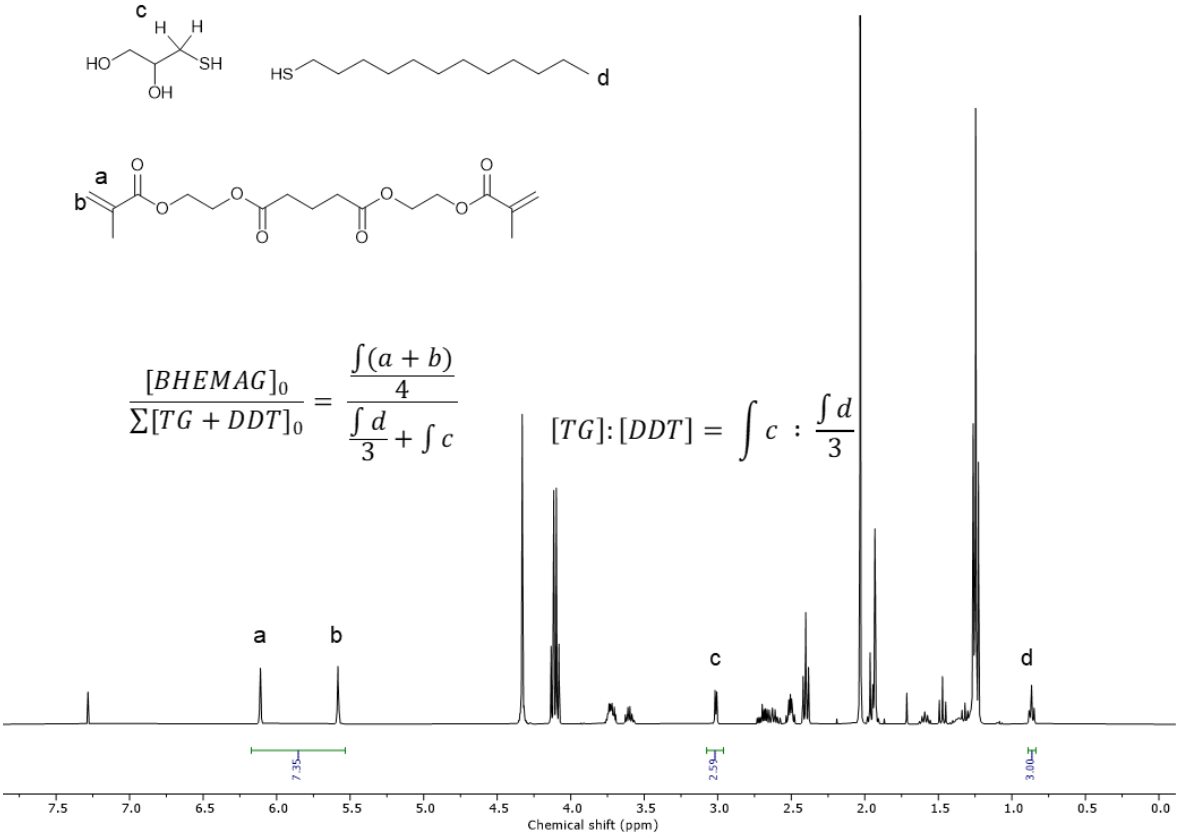

Figure S37 ^{1}H NMR (400 MHz, CDCl3) spectrum obtained from the copolymerisation of BHEMAG with a telogen mixture of DDT and TG prior to thermal initiation (t = 0). The equations provided were used to determine the initial ratio of BHEMAG to the telogen mixture (TG+DDT) ($[BHEMAG]_0/[TG+DDT]_0$) and the overall ratio of TG to DDT within the initial feedstock ($[TG]_0:[DDT]_0$)

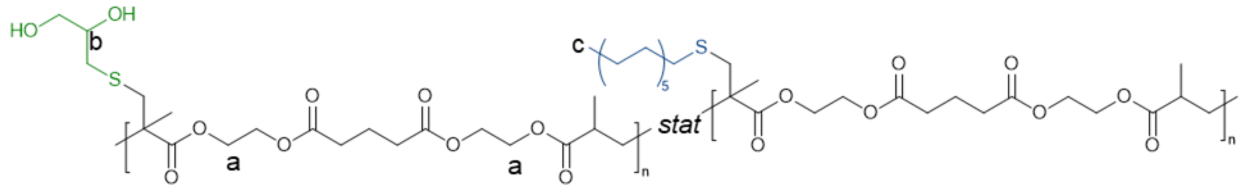

$$\frac{[\mathrm{BHEMAG}]_f}{\sum[TG+DDT]_f} = \frac{\int \frac{a}{4}}{\int \frac{c}{3} + \int b}$$

$$[TG]:[DDT] = \int b : \frac{\int c}{3}$$

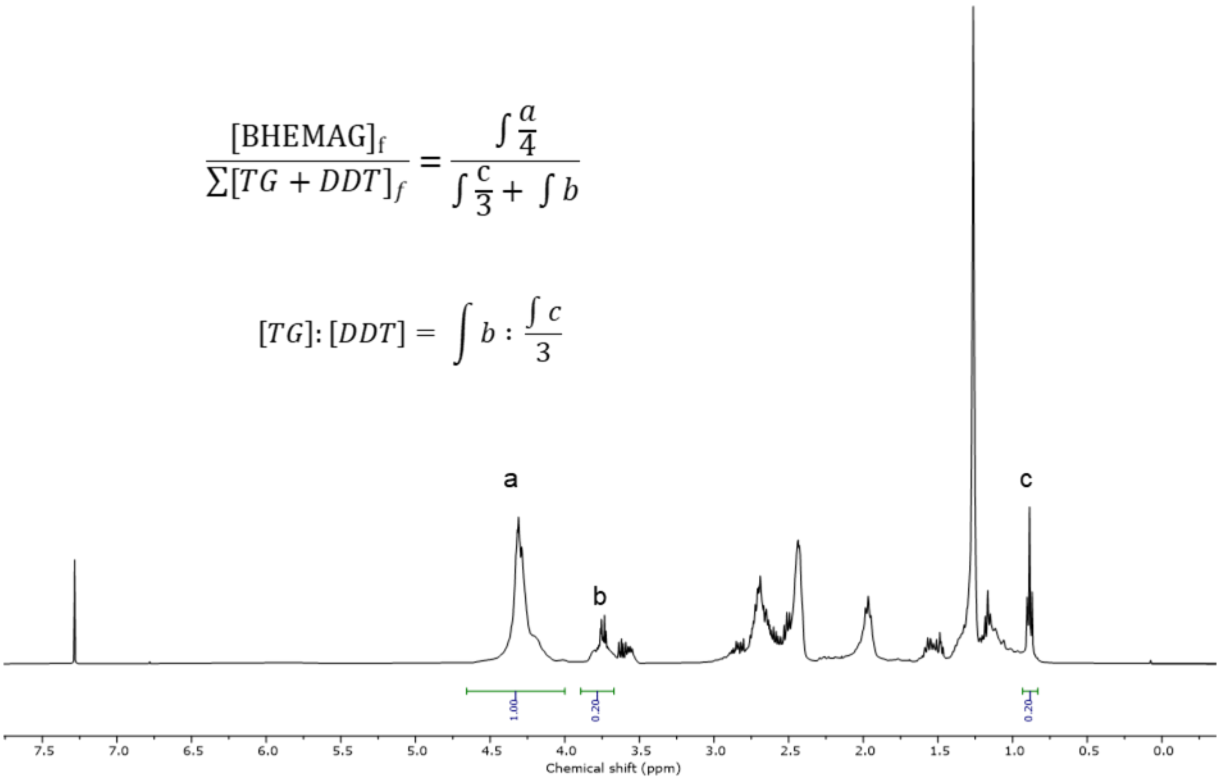

Figure S38 ^{1}H NMR (400 MHz, CDCl3) analysis of *p*([DDT-BHEMAG]$_x$-*stat*-[TG-BHEMAG]$_y$) demonstrating the calculation used to determine the final molar ratio of BHEMAG to total taxogen ([BHEMAG]$_f$/[TG+DDT]$_f$) and the final incorporation of TG to DDT within the repeat unit of the purified polymer ([TG]$_f$:[DDT]$_f$)

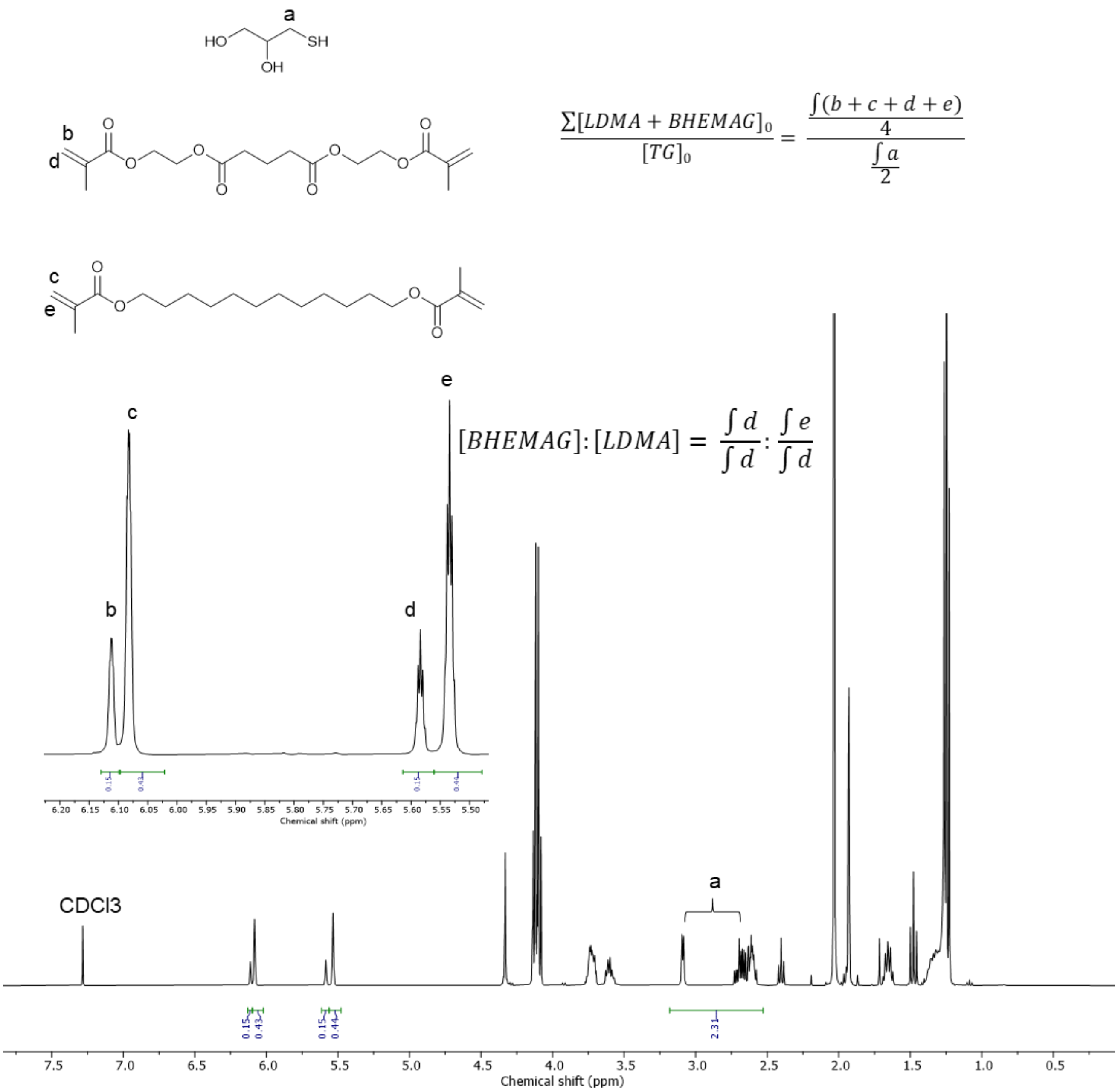

Figure S39 ^{1}H NMR (400 MHz, CDCl3) spectrum obtained from the copolymerisation of a taxogen mixture of LDMA and BHEMAG with TG prior to thermal initiation (t = 0). The equations provided were used to determine the initial ratio of MVT (both BHEMAG and LDMA) to TG ($[BHEMAG + LDMA]_0/[TG]_0$) and the overall ratio of BHEMAG to LDMA within the initial feedstock ($[BHEMAG]_0:[LDMA]_0$)

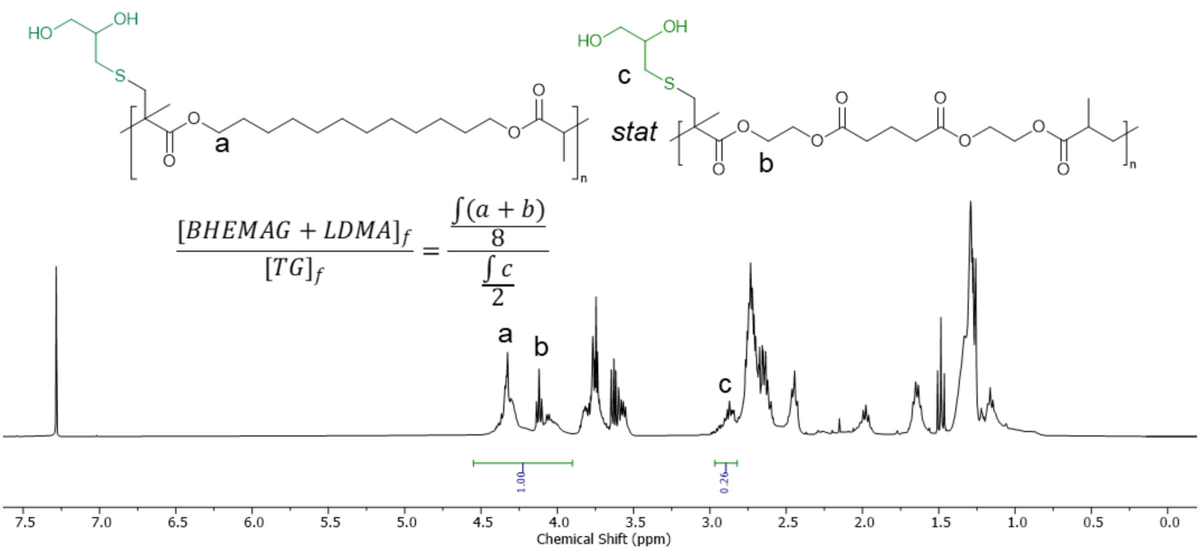

Figure S40 ^{1}H NMR (400 MHz, CDCl3) analysis of - *p*([TG-LDMA]$_x$-*stat*-[TG-BHEMAG]$_y$)demonstrating the calculation used to determine the final ratio of total MVT to telogen within the purified polymer ([MVT]$_f$/[Tel]$_f$)

Table S6 Mass loss data generated post enzyme catalysed hydrolysis of each TBRT homopolymer selected for degradation experiments.

Polymer	Enzyme	Mass Loss (%)
p(ε-caprolactone)	Pseudomonas Lipase	87.1
p(ε-caprolactone)	Pig Liver Esterase	0.0
p(ε-caprolactone)	Porcine Pancreatic Lipase	82.4
p(ε-caprolactone)	Proteinase K	0.0
p(ε-caprolactone)	Protease	0.0
p(ε-caprolactone)	None	0.0
p(LDMA-DDT)	Pseudomonas Lipase	0.0
p(LDMA-DDT)	Pig Liver Esterase	0.0
p(LDMA-DDT)	Porcine Pancreatic Lipase	0.0
p(LDMA-DDT)	Proteinase K	0.0
p(LDMA-DDT)	Protease	0.0
p(LDMA-DDT)	None	0.0
p(LDMA-CHT)	Pseudomonas Lipase	0.0
p(LDMA-CHT)	Pig Liver Esterase	0.0
p(LDMA-CHT)	Porcine Pancreatic Lipase	0.0
p(LDMA-CHT)	Proteinase K	0.0
p(LDMA-CHT)	Protease	0.0
p(LDMA-CHT)	None	0.0
p(LDMA-TG)	Pseudomonas Lipase	12.7
p(LDMA-TG)	Pig Liver Esterase	12.0
p(LDMA-TG)	Porcine Pancreatic Lipase	16.5
p(LDMA-TG)	Proteinase K	19.5
p(LDMA-TG)	Protease	10.7
p(LDMA-TG)	None	0.0
p(BHEMAG-DDT)	Pseudomonas Lipase	13.8
p(BHEMAG-DDT)	Pig Liver Esterase	10.3
p(BHEMAG-DDT)	Porcine Pancreatic Lipase	7.3
p(BHEMAG-DDT)	Proteinase K	0.0
p(BHEMAG-DDT)	Protease	11.3
p(BHEMAG-DDT)	None	0.0
p(BHEMAG-CHT)	Pseudomonas Lipase	14.4
p(BHEMAG-CHT)	Pig Liver Esterase	9.6
p(BHEMAG-CHT)	Porcine Pancreatic Lipase	10.7
p(BHEMAG-CHT)	Proteinase K	0.0
p(BHEMAG-CHT)	Protease	9.2
p(BHEMAG-CHT)	None	0.0
p(BHEMAG-TG)	Pseudomonas Lipase	78.8
p(BHEMAG-TG)	Pig Liver Esterase	39.5
p(BHEMAG-TG)	Porcine Pancreatic Lipase	51.0
p(BHEMAG-TG)	Proteinase K	45.1
p(BHEMAG-TG)	Protease	49.5
p(BHEMAG-TG)	None	0

Table S7 Mass loss data generated post enzyme catalysed hydrolysis of each TBRT mixed telogen copolymer selected for degradation experiments

**Time (days)**	**Mass Remaining (%)**				
	p(BHEMAG-DDT)	p(BHEMAG-DDT)$_{25}$ co - p(BHEMAG-TG)$_{75}$	p(BHEMAG-DDT)$_{50}$ co - p(BHEMAG-TG)$_{50}$	p(BHEMAG-DDT)$_{75}$ co - p(BHEMAG-TG)$_{25}$	p(BHEMAG-TG)
0	100	100	100	100	100
1	100	85	87.3	98.5	72.8
2	97.5	79	85.4	94.4	72
4	95.8	74	82.4	92.8	69.5
7	94.8	58.1	81	91.7	58.6
14	90.9	53.5	77.7	88.9	44.8
21	89.9	49.4	73.9	84.7	37.7
28	89.3	44.4	71.1	81.6	29.1
35	89.7	38.6	70	77.5	18
42	90.1	39.4	71	79.5	14.6

Table S8 Mass loss data generated post enzyme catalysed hydrolysis of each TBRT mixed taxogen copolymer selected for degradation experiments

**Time (days)**	**Mass Remaining (%)**				
	p(LDMA-TG)	p(LDMA-TG)$_{75}$ co - p(BHEMAG-TG)$_{25}$	p(LDMA-TG)$_{50}$ co - p(BHEMAG-TG)$_{50}$	p(LDMA-TG)$_{25}$ co - p(BHEMAG-TG)$_{75}$	p(BHEMAG-TG)
0	100	100	100	100	100
1	94.6	92.6	87.4	79.7	72.8
2	92.6	89.9	84	75.7	72
4	89.1	84.6	80.9	74	69.5
7	85.5	82.4	68.9	64.2	58.6
14	83	81.1	67.6	53.5	44.8
21	81.4	80.4	66.4	52.1	37.7
28	81.1	79.6	65	46	29.1
35	81	77.5	64.2	44.7	18
42	81.3	72.2	61.6	46.9	14.6

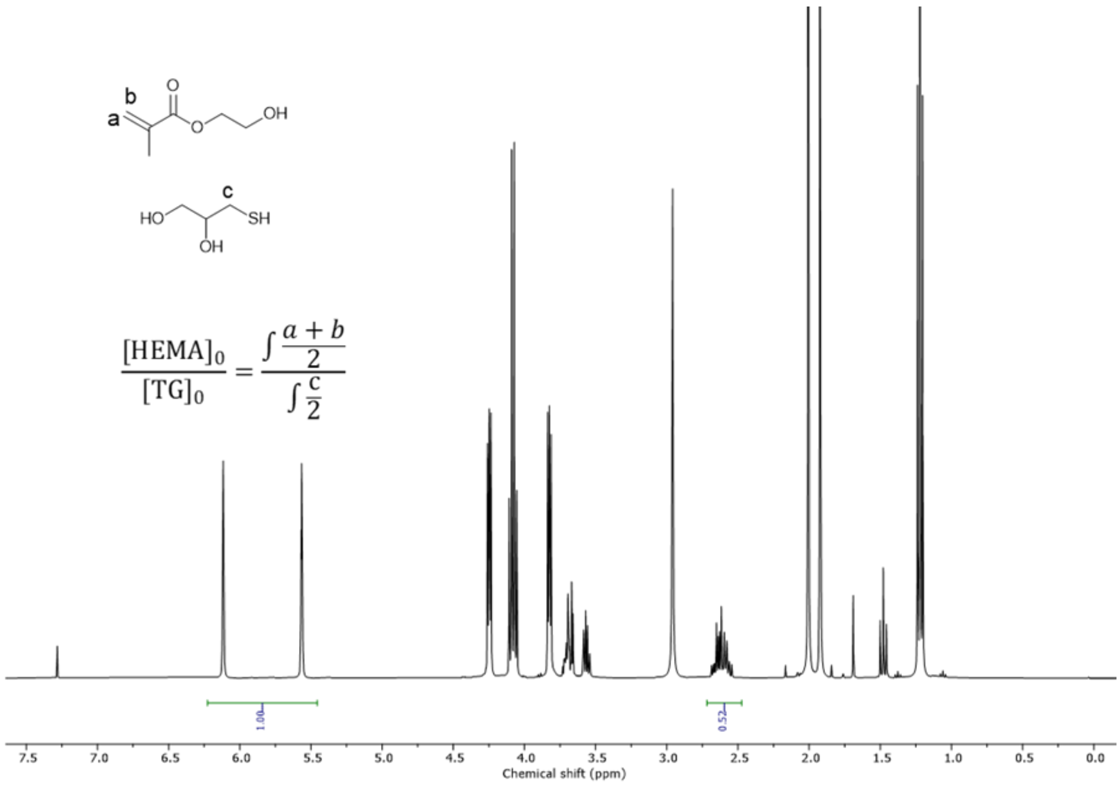

Figure S41 ^{1}H NMR (400 MHz, CDCl3) spectrum obtained from the linear telomerisation of HEMA with TG prior to thermal initiation (t = 0). The equation provided was used to determine the initial ratio of HEMA to TG ($[\mathrm{HEMA}]_0/[\mathrm{TG}]_0$).

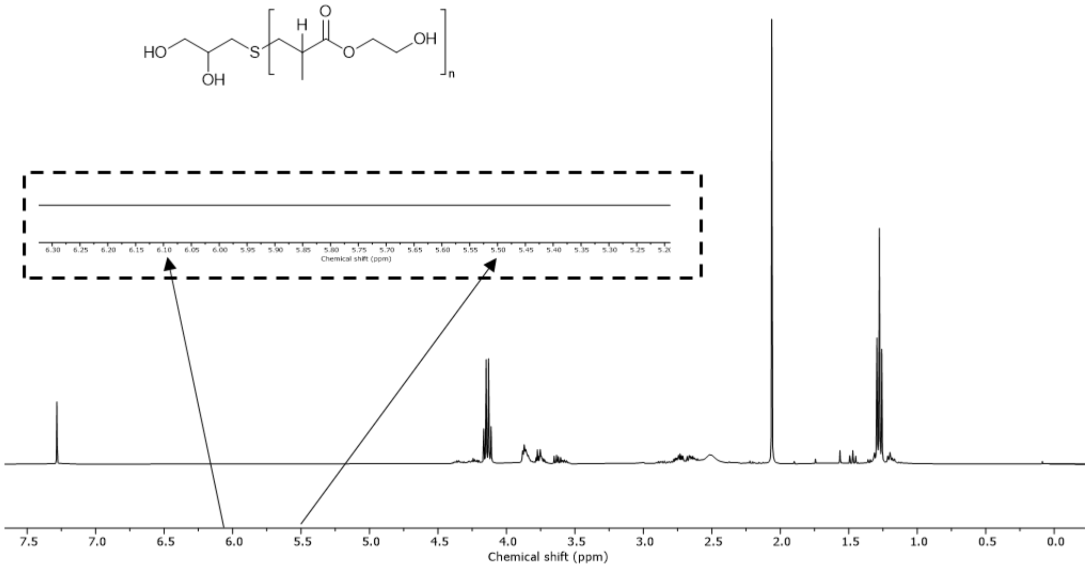

Figure S42 ^{1}H NMR (400 MHz, CDCl3) spectrum obtained from the linear telomerisation of BHEMAT with TG at t = 24 hours. The total disappearance of vinyl signals at chemical shifts 5.50 ppm and 6.20 ppm indicate > 99 % vinyl conversion. Due to the low molecular weight of the polymer analysis was conducted on crude samples.

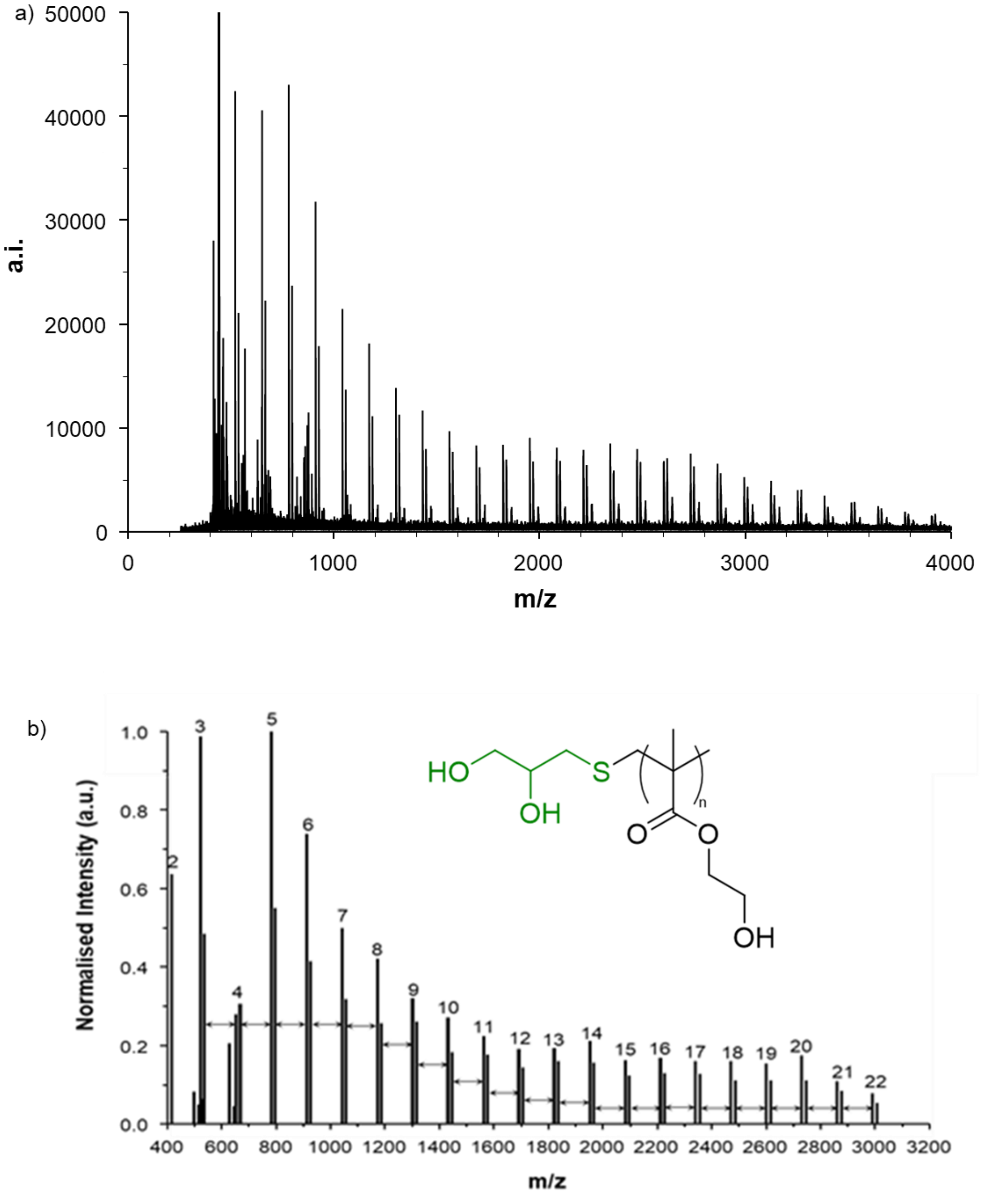

Figure S43 MALDI-TOF mass spectrum obtained from the linear telomerisation with HEMA as the taxogen and 1-thioglycerol as the telogen. a) raw mass spectrum demonstrating the presence of polymeric units with an m/z up to 4000 g mol^{-1}. b) Normalised mass spectrum identifying exclusively p(HEMA) repeat units from the above raw data. Spectrum was acquired in positive reflectron mode.

Table S9 All species detected in MALDI-TOF analysis from the linear telomerisation of HEMA with TG.

DP_n	Mass (m/z)			
	$[M+H]^+$	$[M+H+]^a$	$[M+Na]^+$	$[M+Na]^{+a}$
1	239	255	261	277
2	369	385	391	407
3	499	515	521	537
4	629	645	651	667
5	759	775	781	797
6	889	905	911	927
7	1019	1035	1041	1057
8	1149	1165	1171	1187
9	1279	1295	1301	1317
10	1409	1425	1431	1447
11	1539	1555	1561	1577
12	1669	1685	1691	1707
13	1799	1815	1821	1837
14	1929	1945	1951	1967
15	2059	2075	2081	2097
16	2189	2205	2211	2227
17	2319	2335	2341	2357
18	2449	2465	2471	2487
19	2579	2595	2601	2617
20	2709	2725	2731	2747
21	2839	2855	2861	2877
22	2969	2985	2991	3007

[a] Species corresponding to oxidised sulphur

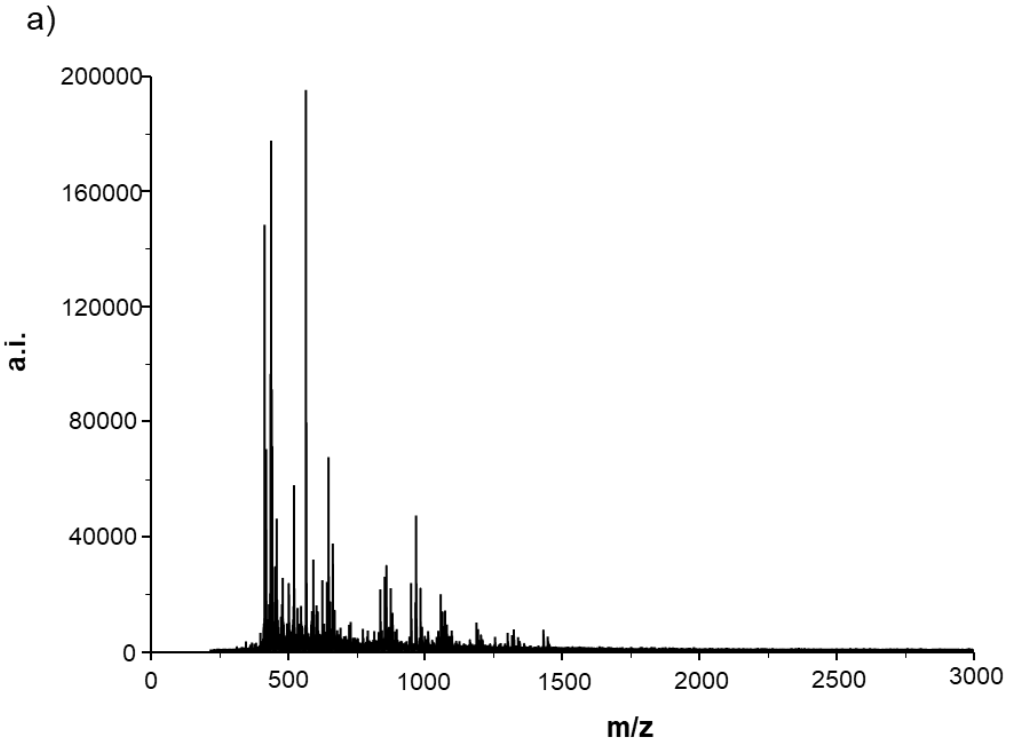

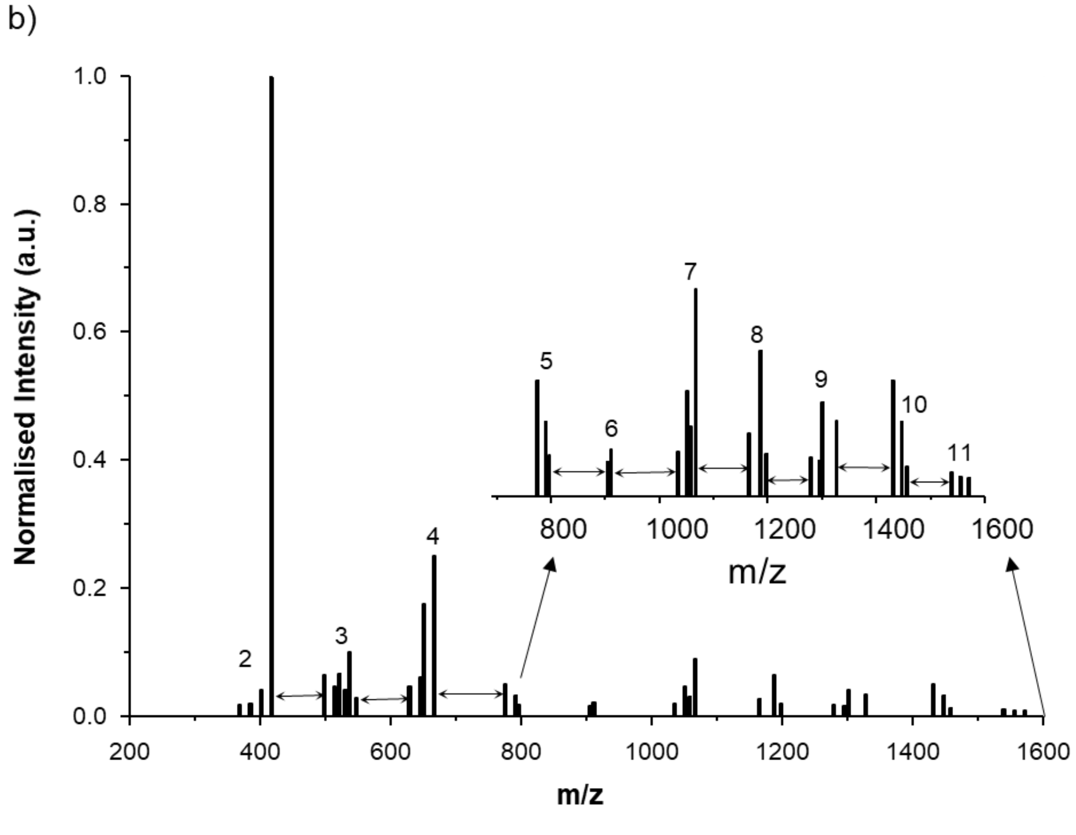

Figure S44 MALDI-TOF mass spectrum obtained from the products of enzyme catalysed hydrolysis of *p*(TG-BHEMAG). a) raw mass spectrum b) Normalised mass spectrum identifying exclusively p(HEMA) repeat units from the above raw data that corresponding to ester group hydrolysis throughout the repeat structure of *p*(TG-BHEMAG). Spectrum was acquired in positive reflectron mode.

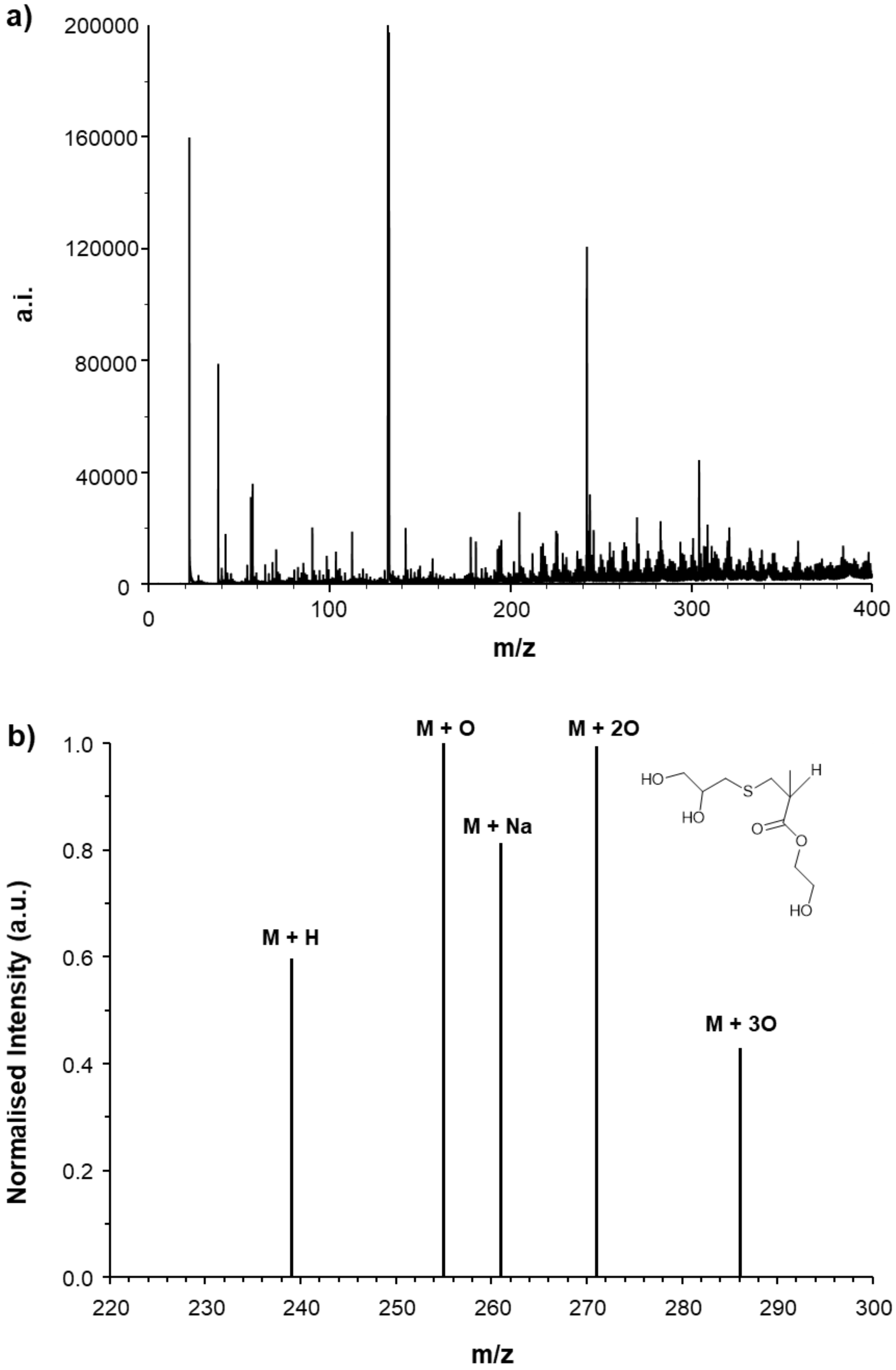

Figure S45 MALDI-TOF mass spectrum obtained from the products of enzyme catalysed hydrolysis of *p*(TG-BHEMAG). Porphyrin matrix utilised here to find DP_1 HEMA-TG adduct. a) raw mass spectrum b) Normalised mass spectrum identifying exclusively p(HEMA) DP_1 Adducts Spectrum was acquired in positive reflectron mode.

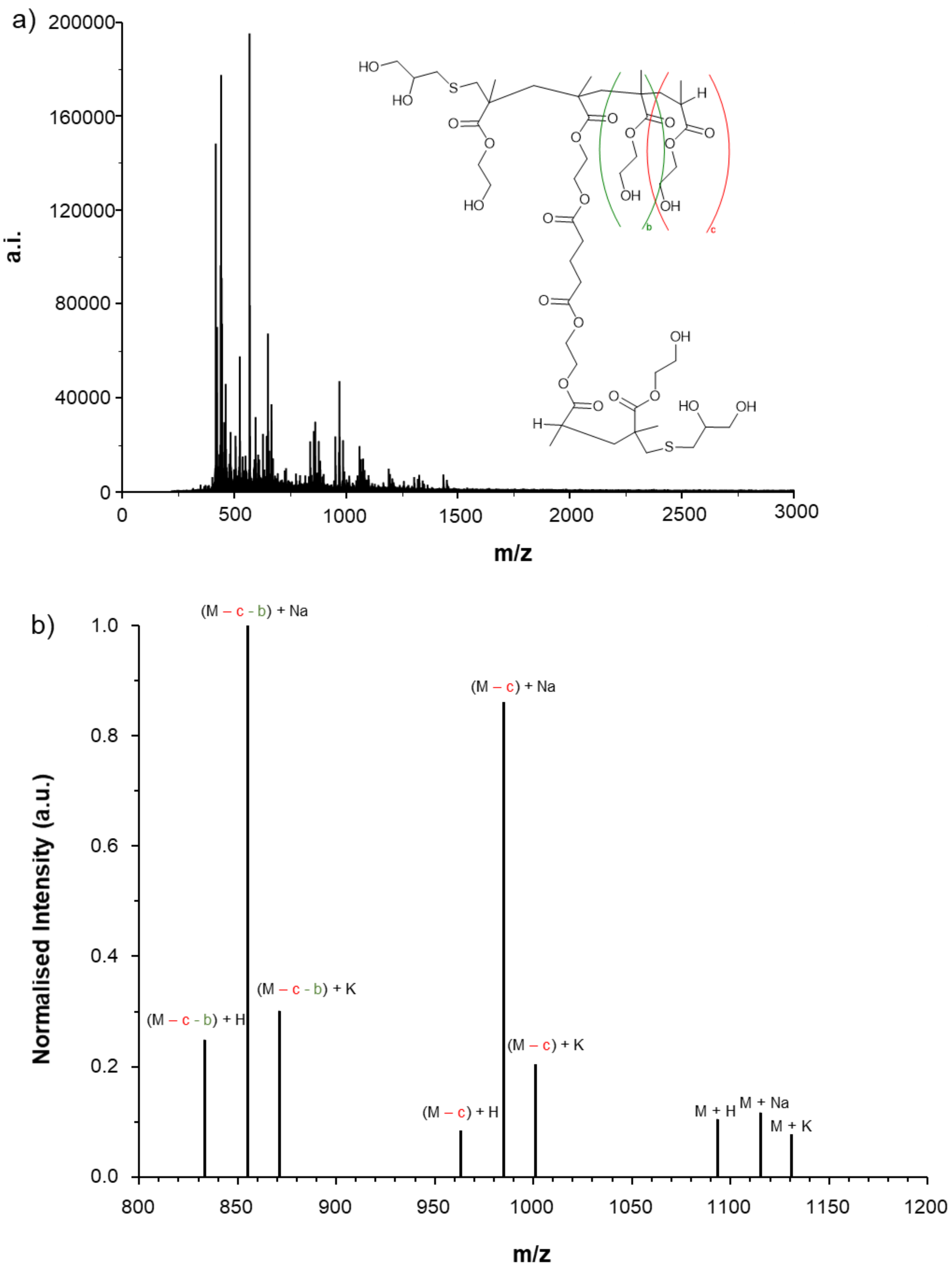

Figure S46 Example MALDI-TOF mass spectrum obtained from the products of enzyme catalysed hydrolysis of *p*(TG-BHEMAG). a) raw mass spectrum b) Normalised mass spectrum identifying exclusively potential alternative products resulting from the partial hydrolysis of *p*(TG-BHEMAG). M represents entire skeletal structure shown above. b represents unit in green. c represents unit in red.

Table S10 All species detected in MALDI-TOF analysis from the enzyme catalysed hydrolysis of *p*(TG-BHEMAG) corresponding to the hydrolysis of glutaric esters within the polymer repeat structure, thus indicating the formation of structures identical to DP_x *tel*(HEMA-TG)

DP_n	Mass (m/z)			
	$[M+H]^+$	$[M+H+]^a$	$[M+Na]^+$	$[M+Na]^{+a}$
1	238.86	254.88	261.08	277.98
2	367.16	-	391.32	407.15
3	499.11	516.08	521.07	537.33
4	629.01	645	651	667.03
5	-	775.26	781.4	797.22
6	-	905.25	911.34	927.34
7	-	1035.35	-	1057.35
8	1149.43	1165.41	-	1187.53
9	1278.15	1295.21	-	1317.4
10	-	1425.67	1431.59	1447.59
11	1539.58	1555.47	-	1577.53

[a] Species corresponding to oxidised sulphur
